# Medical gas plasma modifies Nrf2 signaling in diabetic wound healing

**DOI:** 10.1016/j.jare.2025.04.020

**Published:** 2025-04-16

**Authors:** Anke Schmidt, Lea Miebach, Can Bagli, Liane Kantz, Steffen Emmert, Thomas von Woedtke, Sander Bekeschus

**Affiliations:** a ZIK Plasmatis, Leibniz Institute for Plasma Science and Technology (INP), a member of the Leibniz Health Research Alliance, Felix-Hausdorff-Str. 2, Greifswald 17489, Germany; b Department of Dermatology and Venereology, University Medical Center Rostock, Strempelstr. 13, Rostock 18057, Germany; c Institute of Hygiene and Environmental Medicine, Greifswald University Medical Center, Sauerbruchstr., Greifswald 17475, Germany

**Keywords:** Cytokines, Diabetes mellitus, Nrf2, Plasma medicine, Reactive species, Redox regulation

## Abstract

**Introduction:**

Diabetes mellitus is a chronic disease that can disrupt physiologic wound healing. Medical gas plasma technology produces therapeutic reactive species that support wound healing.

**Objective:**

Previous studies have shown that increasing the transcriptional activity of the redox regulator nuclear factor erythroid 2-related factor 2 (Nrf2) in diabetic models can improve insulin sensitivity, reduce blood glucose levels, and ameliorate diabetic complications. However, the therapeutic potential and mechanisms of action of gas plasma have not been addressed in this context.

**Methods:**

Full-thickness dermal ear wounds were created in a preclinical mouse model of type II diabetes and compared with a native wild-type strain of C57BL/6 mice. First, the formation of reactive species in the plasma gas phase was determined by optical emission spectroscopy. Second, qPCR, protein expression, and inflammation analysis by cytokine secretion were performed to confirm the transcriptional results. Finally, qPCR and cytokine profiling were conducted to measure the effects of gas plasma in patient wound samples.

**Results:**

Repeated in vivo treatment with medical gas plasma supported wound healing, e.g., re-epithelialization, in both sexes. Gas plasma-stimulated changes in Nrf2 signaling associated with downstream targets were supported by the evidence of impaired wound healing in Nrf2 knockout mice. In addition, gas plasma treatment significantly affected inflammation by modulating local and systemic cytokine levels. *In vivo*, treatment of human diabetic wounds underscored the involvement of Nrf2 signaling in protecting against oxidative stress, as assessed by qPCR. The cytokine signature of human diabetic wounds outlined different response patterns among patients after a single exposure, while inflammatory mediators were consistently reduced after repeated plasma treatment.

**Conclusions:**

The present finding of accelerated wound healing by the Nrf2 activator underlines the high potential of medical gas plasma therapy in non-diabetic and diabetic wound healing.

## Highlights

• Gas plasma treatment improved wound healing in both wild-type and diabetic mice.
• Wound healing was defective in Nrf2 knock-out mice and could not be fully rescued by gas plasma exposure.
• Pro-wound healing processes were more pronounced in female compared to male mice.
• In patient-derived wound samples, we found that gas plasma exposure regulates inflammatory processes associated to Nrf2.

## Graphical abstract

**Figure:**
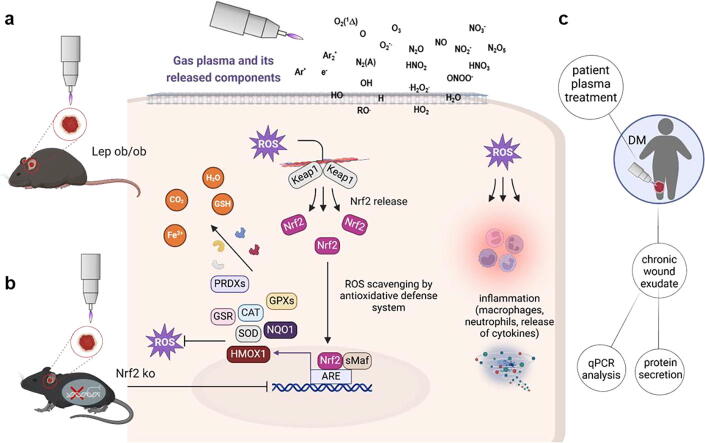

## Introduction

Diabetes mellitus type II is a chronic disease characterized by insulin resistance, insulin deficiency, and impaired glucose homeostasis [1]. Type II diabetes, along with its associated complications, such as neuropathy, nephropathy, retinopathy, and the resulting risk of cardiovascular disease [[2], [3]], is a global health problem contributing to the increasing burden of mortality. In contrast to autoimmune type I diabetes, type II diabetes is the most common form of diabetes, and its prevalence is increasing worldwide [4]. Type II diabetes is estimated to affect 537 million people worldwide and is expected to rise to 783 million by 2045 [5]. Diabetic foot ulcers are the most common type of diabetic wound in both types of diabetes, with a high recurrence rate of up to 50 % [6]. Wound healing in people with diabetes is complex and challenging because several factors interfere with normal healing [7]. Due to the impaired ability of diabetic individuals to produce and respond to growth factors, cytokines, and other signaling molecules essential for wound healing, diabetic wounds are characterized by delayed healing, chronic inflammation, and increased susceptibility to infection. These include increased oxidative stress, impaired immune function, decreased angiogenesis, and altered extracellular matrix composition [6]. The choice of treatment modality for diabetes depends on the specific needs of the individual and the particular characteristics of the wound. Based on some relevant human characteristics, several rodent models have been used to study diabetes-related delayed wound healing [8]. Here, we used a model of type II diabetes, which is characterized by leptin deficiency leading to persistent obesity [9] with several metabolic alterations, including hyperlipidemia, temperature regulation defects, and decreased physical activity [10].

The pathophysiological production of reactive oxygen and nitrogen species (collectively ROS) in diabetes plays a significant role in the development and progression of diabetes-related complications, including insulin resistance, β-cell dysfunction, inflammation, vascular complications, and oxidative damage [11]. Management of ROS is critical to prevent these features and improve health outcomes. Gas plasma is a partially ionized gas containing various reactive molecules, including charged particles, ions, electrons, and ROS [12]. These reactive species can directly interact with biological tissues and cells and modulate cellular signaling pathways through various mechanisms, including cell proliferation and migration stimulation, enhanced extracellular matrix remodeling, and increased angiogenesis [13]. Gas plasma has emerged as a promising treatment modality for acute wounds due to its ability to promote wound closure [14]. While several preclinical studies have been successfully applied in rodent models [[15], [16], [17], [18]],clinical plasma therapy has also shown beneficial effects in patients with chronic wounds and ulcers [[19], [20], [21]], as well as in patients with chronic inflammatory skin diseases [22]. Some studies found that gas plasma treatment significantly reduced bacterial colonization in chronic diabetic foot ulcers and improved wound healing rates compared to standard care alone [23]. Consequently, it is hypothesized that gas plasma modulates several cellular processes related to redox signaling and may help target various specific wound healing pathways [14]. A significant advantage is that gas plasma treatment is non-invasive, painless, and safe, with no adverse effects reported in clinical trials [24], making it suitable for outpatient use.

Dysregulation of the nuclear factor erythroid 2-related factor 2 (Nrf2) signaling has been implicated in several diseases, including diabetes. The Nrf2 signaling pathway is a critical mediator in the cellular response to oxidative stress [[25], [26]] and inflammation [27]. By regulating the expression of antioxidant and detoxification enzymes, Nrf2 helps maintain cellular redox balance and modulates inflammatory responses [28]. This dual role makes it an attractive therapeutic intervention target for chronic inflammatory diseases. However, the mechanism of plasma-mediated action on diabetic wound healing is not fully understood. In this work, we demonstrate the critical role of Nrf2 signaling in gas plasma-mediated diabetic wound healing using type II diabetes-induced wild-type and Nrf2-knockout mouse strains. We showed that oxidative stress-responsive targets (e.g., Nrf2) were simultaneously regulated in gas plasma-assisted wound healing. In addition, gas plasma-enhanced healing was paralleled by altered chemokine and cytokine release. Human samples isolated from gas plasma-treated diabetic wounds and analyzed for key Nrf2 targets confirmed the above findings.

## Methodology

### Gas plasma device and optical emission spectroscopy

The argon plasma jet kINPen MED (neoplas med, Greifswald, Germany) operated at 1 MHz and 2–6 kV with argon gas (purity 99.9999; Air Liquide, Bremen, Germany). The device has been approved as a class IIa medical product in Germany and Europe since 2013 [29]. Optical emission spectroscopy (OES) was performed to evaluate the excited ROS generated by the kINPen Med operated with argon at 5 slm. Briefly, an optical fiber was placed below the plasma effluent and connected to the optical emission spectrometer (0.7 nm of spectral resolution using an AvaSpec-3648-USB2 device; Avantes, Germany). The distance between the kINPen nozzle and the fiber holder was 2 cm.

### Reactive species profiling

Reactive species formation was measured using fluorescent and colorimetric sensors used in liquid analysis. Briefly, 100 µl of PBS was exposed to gas plasma (2 slm) for 1 s, 5 s, 10 s, and 15 s. Evaporation was compensated by a predetermined volume of double-distilled water to maintain *iso*-osmolarity. 2-(4-Carboxyphenyl)-4,4,5,5-tetramethylimidazoline-1-oxyl-3-oxide potassium salt (cPTIO; Thermo Fisher, Germany) was added as a scavenger for nitric oxide (NO^.^) at a concentration of 100 µM. Relative assessment of NO^.^ was performed using 4-amino-5-methylamino-2′,7′-dichlorofluorescein (DAF-FM, while aminophenyl fluorescein (APF; both Thermo Scientific, Germany) was used to measure peroxynitrite (ONOO^–^), hydroxyl radical (^.^OH), and hypochlorous acid (HOCl) deposition. Before treatment, the probes were added to PBS at a final concentration of 5 µM. The fluorescence signal was determined immediately after treatment at λ_ex_ 485 nm and λ_em_ 525 nm using a microplate reader (Infinite F200 Pro; Tecan, Switzerland). The respective probes were added to cell-free hydrogels (BME; R&D Systems, Germany) prior to polymerization to evaluate ROS penetration in tissue-like matrices. Images were acquired in fluorescence channels at λ_ex_ = 475 nm and λ_em =_ 525 nm using a 20x air objective (NA = 0.4, Zeiss, Germany) in spinning disk confocal mode over 13 z-stacks with a 200 µm plane spacing for each well (Operetta CLS; PerkinElmer, Germany). Algorithm-driven unsupervised image analysis was performed to assess the oxidation of the embedded ROS sensors based on fluorescence signals. In addition, nitrite (NO_2_^–^) was quantified in plasma-treated PBS using the *Griess* assay (Cayman Chemicals, Germany) according to the manufacturer's instructions. Absorbance was measured at 540 nm using a multimode plate reader (Infinite M200 Pro; Tecan, Switzerland). Concentrations were calculated using appropriate standard curves.

### Animals, wounding, and medical gas plasma treatment

The mouse strains B6.Cg-Lepob/J (Lep^ob^ or diabetic), B6.129X1-Nfe2l2tm1Ywk/J (accession number: 017009, Nrf2 ko) and wild-type C57BL/6J (Charles River Laboratories, Germany) at 8 weeks of age were used for wound healing studies under protocols approved by the local ethics committee (approval code: 7221.3–1-044/16) in accordance with the guidelines and NIH Guide for the Care and Use of Laboratory Animals. A 12-hour light/dark cycle was maintained. Free access to food and water was provided (Central Core and Research Facility of Laboratory Animals, Greifswald University Medical Center, Germany). Baseline blood glucose levels in mice vary depending on strain, age, and sex, but some general ranges were between 62–175 mg/dL in the wild-type C57BL/6J and Nrf2 knockout mouse models, as opposed to blood glucose levels in the range of approximately 300 to 600 mg/dL reflecting the hyperglycemic state characteristic of the B6.Cg-Lepob/J model. Both ear wounds were prepared as previously described [18]. Gas plasma treatment of ear wounds approximately 4 mm [2] in size was performed with the tip of the plasma jet effluent connected to the tissue (i.e., conductive mode [30]) at a distance of 8  mm using a reusable spacer. Mice were divided into eight groups for females and males (four to five mice from each model per group). Experiments were performed for 9 days with four sessions of 10 s plasma treatment per wound or 20 days with six sessions of 10 s plasma treatment per wound and compared with corresponding untreated controls (Table 1). Wound size, epidermal thickness, and granulation were measured on the day of wounding (d0) and every third day after that. The camera system TIVITA Tissue and the camera-specific software TIVITA Wound Suite 1.0 (Diaspective Vision, Germany) were used to analyze the wound size in real time. Gas plasma exposure distance and treatment time are based on our previous experience using murine models of wound healing and clinical data on the kINPen argon plasma jet [14].

**Table 1:** Overview of the experimental groups, time of sacrifice after wounding, the treatment regimes, and sex (d, day; s, seconds).

**mouse model**	**groups**	**day of tissue collection**	**treatment regime**	**wounds in females ♀**	**wounds in males ♂**
B6.Cg-Lepob/J	ctrl	d9	0 s	8	8
(diabetic)	plasma		every 3rd day over 9 day (4x), 10 s	8	8
	ctrl	d20	0 s	10	8
	plasma		every 3rd day over 20 days (6x), 10 s	10	8
B6.129X1-Nfe2/2/J	ctrl	d9	0 s	10	10
(Nrf2 ko)	plasma		every 3rd day over 9 day (4x), 10 s	10	10
	ctrl	d20	0 s	8	8
	plasma		every 3rd day over 20 days (6x), 10 s	8	8
C57BL/6J	ctrl	d9	0 s	10	8
(B6)	plasma		every 3rd day over 9 day (4x), 10 s	10	8
	ctrl	d20	0 s	8	10
	plasma		every 3rd day over 20 days (6x), 10 s	8	10

### Patients-derived diabetic wound material

Written informed consent was obtained from all patients, in line with the approval of the local ethics committee (approval number: A 2021–0230 Rostock; BB029/21 Greifswald). Patients with chronic wounds (diabetic foot syndrome) signed a written informed consent prior to participation in the study. To collect primary diabetic wound exudate, the wound dressing was removed, rinsed with sterile saline (0.9 %), and collected with sterile swabs. During debridement, the wound was mechanically cleaned with a disinfectant solution (e.g., Octenisept, Lavasorb, Lavanox, or Prontosan), and a sterile curette was used to remove fibrin deposits, small necrotic deposits, and macerated tissue (softened wound edge) to refresh the wound edges. After final wound disinfection, gas plasma treatment was performed by moving the plasma effluent slowly over the wound in a meandering motion at approximately 10 s/cm^2^. The dry wound surface was moistened with physiological saline using FLOQ swabs. The swabs were collected in tubes containing 500 µL of RNA lysis buffer (Bio&Sell, Germany), stored on ice, and processed immediately. For qPCR analysis, diabetic wound exudates were obtained from diabetic patients undergoing single (n = 5) or repeated (n = 4) medical gas plasma treatment. In addition, untreated wound exudate (n = 9) was isolated from each patient prior to plasma treatment (Table S3). Repeated plasma treatment was administered once a week; after five weeks, samples were collected and divided into the repeated plasma group. For the bead-based sandwich immunoassay, wound exudates from untreated (before plasma treatment) and plasma-treated wounds were collected in 100–500 µl of PBS (n = 9).

### Quantitative polymerase chain reaction (PCR) and WES analysis

Wounded ear tissues were collected after animal sacrifice on either day 9 or day 20 post-injury (Table1). For analysis, fresh tissue from one ear was snap-frozen in liquid nitrogen and stored at −80 °C. Mouse and human samples were homogenized in RNA lysis buffer (Bio&Sell, Germany) for gene expression analysis using a FastPrep-24 5G homogenizer (MP Biomedicals, Germany). In addition, mouse tissues were homogenized in RIPA buffer containing protease and phosphatase inhibitors (cOmplete Mini, phosSTOP, PMSF; Sigma-Aldrich, Germany) for protein validation experiments. To assess mRNA levels by quantitative PCR (qPCR), 1  μg of RNA was transcribed into cDNA, and qPCR was performed in duplicate using SYBR Green I Master (Roche Diagnostics, Switzerland) and gene-specific primers (BioTez, Germany; Table S1 for mouse model and Table S2 for human samples). The unaffected housekeeping genes *GAPDH* and *RPL13A* were used as normalization controls. Gene expression was analyzed using the 2^-△△CT^ method. Quantitative changes in PCR were confirmed on the protein level, including targets of the Nrf2-pathway (e.g., Nrf2, Nqo1, and HO-1) and antioxidant response (e.g., Cat, Sod1, and GSR). Gapdh was used as a housekeeping protein (all antibodies from Cell Signaling Technology, Germany). Simple Western technology (WES) was applied using fully automated capillary electrophoresis and immunoassay detection according to the manufacturer's instructions (ProteinSimple, Germany). Protein expression was quantified using ProteinSimple software (Compass, Germany) and expressed as fold change relative to the corresponding untreated control.

### Bead-based cytokine and chemokine profiling in blood serum and protein lysates

Blood was collected retrobulbar in EDTA tubes on days 0, 9, and 20 and centrifuged, and the serum supernatant was stored at −80 °C until use. Inflammatory secretion profiles in blood serum and protein lysates from diabetic and Nrf2-ko mice were measured using multiplex detection technology according to the instructions (LegendPlex; BioLegend, The Netherlands). Briefly, the bead-based sandwich immunoassay was measured by flow cytometry (CytoFLEX LX; Beckman-Coulter, Germany) targeting granulocyte–macrophage colony-stimulating factor (GM-CSF); tumor necrosis factor (TNF) α; interferons (IFN) α, β and γ; four chemokines (monocyte chemotactic protein (MCP1 or CCL2), chemokine ligand 5 (RANTES or CCL5), C-X-C motif chemokine 1 (KC or CXCL1), and C–X–C motif chemokine 10 (IP10 or CXCL10), and four interleukins (IL1β, IL6, IL10, and IL12p70). LegendPLEX data software (BioLegend, The Netherlands) was used to quantify cytokine levels.

### Histology, immunohistochemistry, and immunofluorescence analysis

On days 9 and 20 after wounding, the wound tissue of one ear was fixed in 4 % paraformaldehyde (Sigma-Aldrich, Germany) overnight. Paraffin-embedded tissue blocks were sectioned at 5  µm using a microtome and stained with hematoxylin and eosin (H&E; Carl-Roth, Germany). Collagen fibers were stained with picro-sirius red (PSR 80; Sigma-Aldrich, Germany) as described before [31]. For immunohistochemistry, tissue samples were stained with primary antibodies and visualized with SignalStain boost IHC detection reagents (all Cell Signaling Technology, Germany). For immunofluorescence microscopy analysis of several proteins in tissues, samples were washed, permeabilized with Triton X-100 (0.01 % in PBS; Sigma-Aldrich, Germany), and incubated with primary antibodies against Nrf2 and TGFβ (all Cell Signaling Technology, Germany) followed by staining with secondary fluorescence antibodies (Life Technologies, Germany). Stained sections were counterstained with DAPI and mounted on glass slides using VectaShield (Biozol, Germany) before fluorescence microscopy was performed using an Axio Observer Z.1 (Zeiss, Germany).

### Statistical analysis

All experiments were performed on tissues from a minimum of five wounds per group. *In vitro* assays were repeated independently in triplicates. Graphing and statistical analysis were done using *prism* 9.51 (GraphPad Software, USA) and presented as values are mean + S.D. unless otherwise indicated. Student's *t*-test was used for comparison between two groups and two-way analysis of variance (ANOVA) for multiple group comparison (*p < 0.05, **p < 0.01, or ***p < 0.001). All significance values of the qPCR data are shown in the graphs only if they are below or above 0.67 and 1.5 (dashed lines), respectively. Some schemes were designed using a commercial license of biorender.

## Results

### Gas plasma promotes wound healing in diabetic mice

**Fig. 1:**
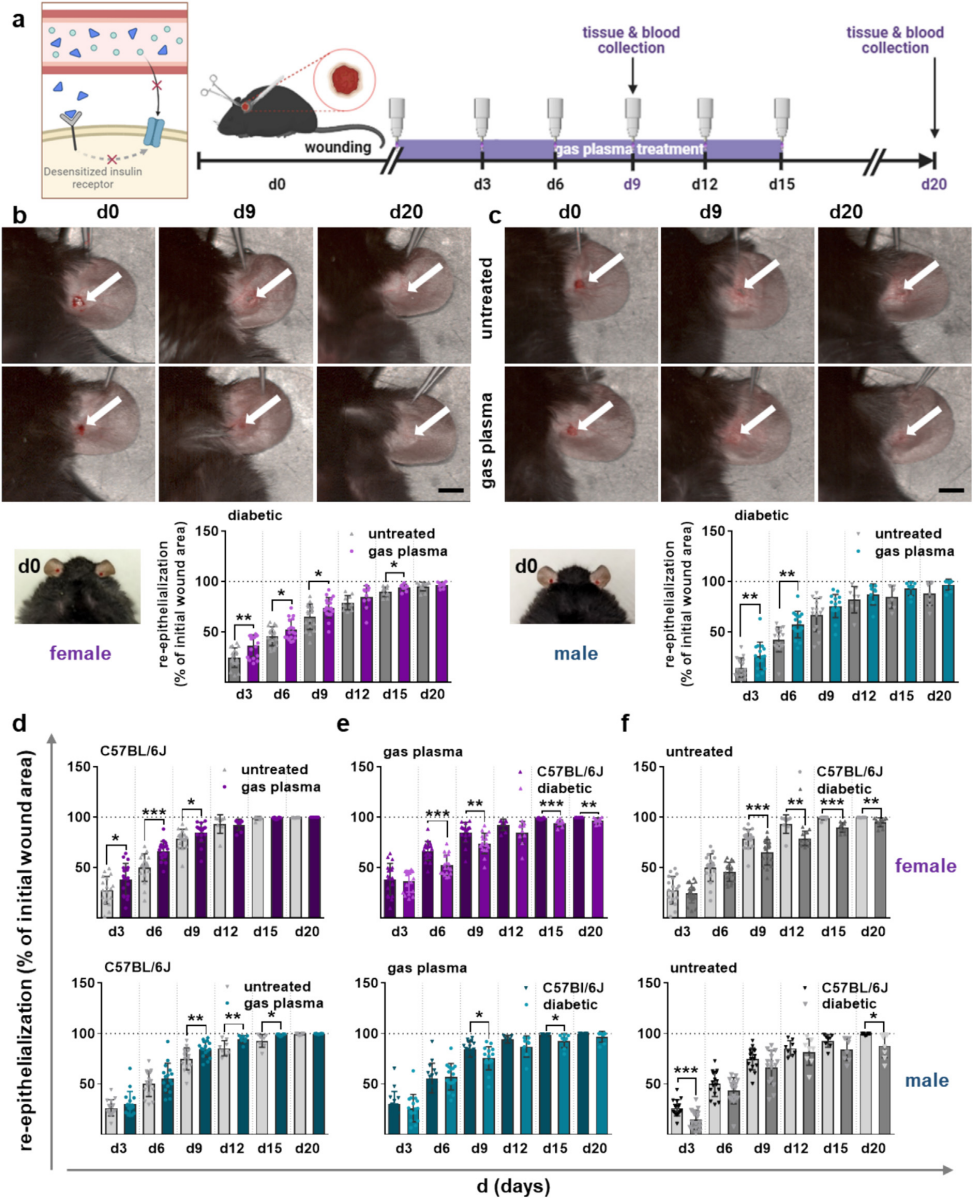

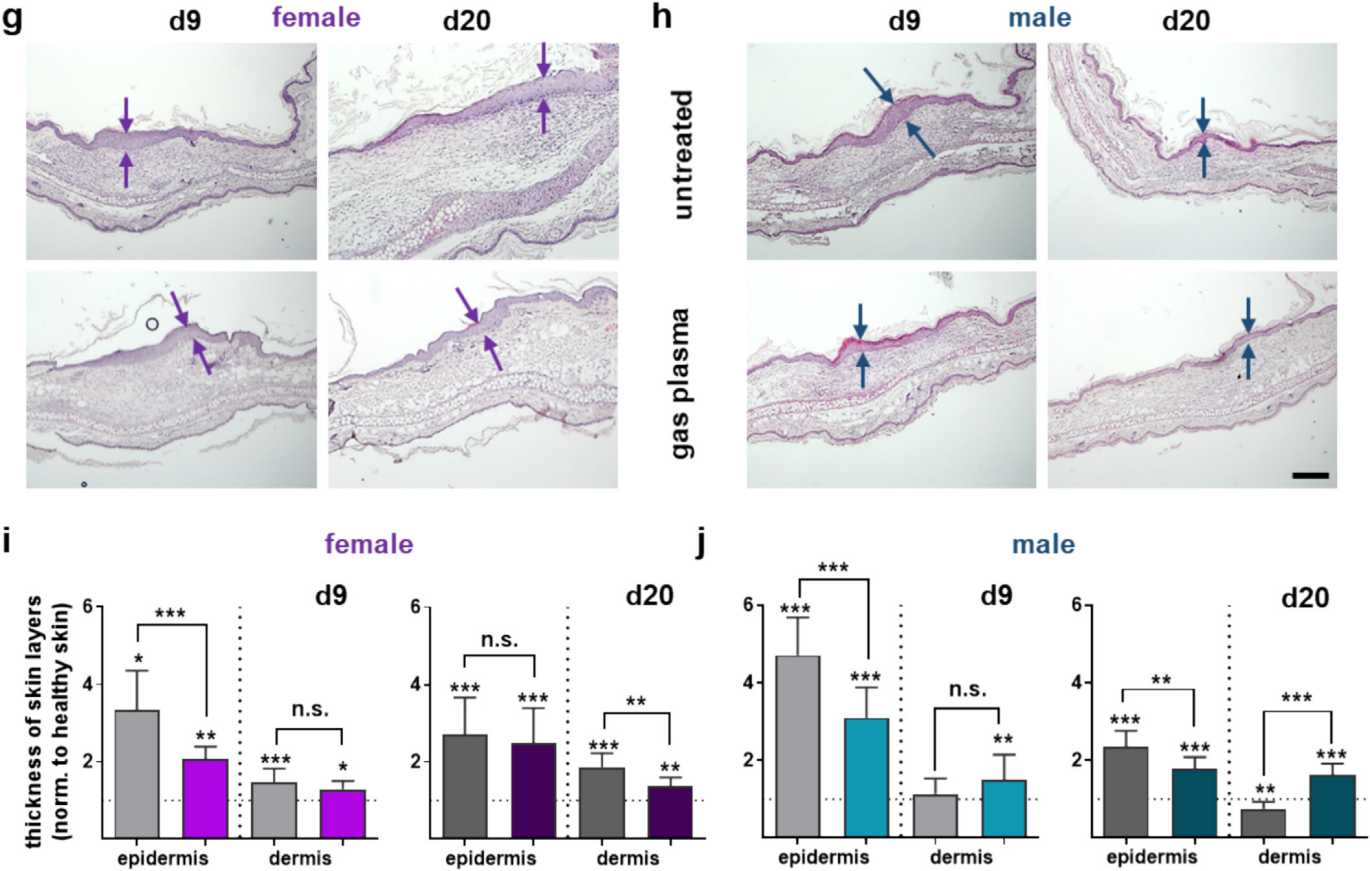
Wound closure in diabetic mice was shortened by repeated gas plasma treatment. (a) Schematic of type II diabetes and schematic time course of gas plasma treatment in diabetic mice illustrating ear wound generation and treatment regimen. (b-c) Representative images of wound healing on days 0, 9, and 20 in untreated controls (ctrl, top panel) and gas plasma-treated (bottom panel) females (b) and males (c). The rate of wound closure (re-epithelialization) is plotted as the percentage reduction of the original wound area over time for treated mice when compared to the untreated controls in diabetic females (b) and diabetic males (c); for (d) gas plasma-treated non-diabetic wild-type C57BL/6J mice when compared to their untreated controls, (e) gas plasma-treated diabetic mice when compared to C57BL/6J mice, and (f) untreated diabetic mice when compared to C57BL/6J mice. Individual scatter is shown for each group: top pink panel for females and bottom cyan panel for males. (g-h) Representative images of hematoxylin and eosin (H&E) staining after wounding illustrate the stages of re-epithelialization and histological changes with the migration of skin cells into the wound bed at d9 and d20 in females (g) and males (h). (i-j) Thickness quantification of epidermis and dermis at both endpoints in gas plasma-treated and untreated wounds (gray columns) compared to healthy (unwounded) skin (set to 1) in females (i, pink) and males (j, cyan). Data are presented as mean ± S.D.; *p < 0.05, **p < 0.01, and ***p < 0.001 compared to controls (ns = not significant); scale bars are 1 cm (b, c); 200 µm (d20 in g) (100  µm (d9 in g, h); females and males were used at d9 and d20 (n ≥ 4).

Considering gender differences and heterogeneity [32], both ear wounds in females and males were treated with gas plasma for 10 s every third day for 9 (d9; on d0, d3, d6, d9) and 20 days (d20, on d0, d3, d6, d9, d12, d15) and compared to untreated controls (Fig. 1**a**). Representative images show wounds in untreated (top panels) versus gas plasma-treated mice in females (Fig. 1**b**) and males (Fig. 1**c**) after wounding (d0) and at both endpoints (d9 and d20). Each analysis interval showed accelerated wound healing in the gas plasma-treated wounds compared to the untreated wounds, which was significant, as indicated. Almost complete wound closure and maturation of the epidermal and dermal layers were achieved in two to three weeks (graphs, Fig. 1**b-c**). In addition, we compared healing responses in terms of wound closure time and re-epithelialization in both sexes with all possible treatment and control groups, including the non-diabetic wild-type strain C57BL/6J. Gas plasma treatment shortened the time of re-epithelialization in females (top panel, pink) and males (bottom panel, cyan) in non-diabetic wounds (Fig. 1**d**). Furthermore, impaired wound closure was observed in diabetic mice receiving gas plasma therapy compared to wild-type mice (Fig. 1**e**), confirming previous work in type I diabetic mice [[33], [34]]. Similarly, wound closure rates were higher in non-diabetic untreated controls than in diabetic mice (Fig. 1**f**).

By histologic observation during the healing process, we demonstrated that the dermal defects were gradually closed by newly formed granulation tissue covered by the epidermis. Inflammatory cell aggregates or swollen, multinucleated macrophages, as standard features of injury-induced inflammation, were not detected in hematoxylin and eosin (H&E)-stained tissues in any of the treatment groups, suggesting normal, physiological healing processes in both females (Fig. 1**g**) and males (Fig. 1**h**). We quantified the differences between the experimental groups in the thickness of the epidermis and the dermal compartments with granulation tissue in the wound areas. The wound areas were readily identifiable, as thickening of the epidermis and apparent changes in the dermal granulation area were observed in the wound zone of all experimental groups compared to healthy skin tissue. With a statistically significant difference at both endpoints, gas plasma-treated mice showed significantly reduced epidermal thickness at d9 and dermal thickness at d20 in female mice compared to untreated wounds. In addition, the thickness of the newly formed epidermal layer was still higher than in healthy skin tissue but comparable in both experimental groups at day 20 after wounding (Fig. 1**i**). In males, we found a higher proportion of re-epithelialized wounds and granulation tissue along with a slightly lower epidermal thickness after injury at both endpoints compared to untreated wounds (Fig. 1**j**), indicating a slightly different healing process compared to females.

### Profiling of nitric oxide and secondary nitrogen species

**Fig. 2:**
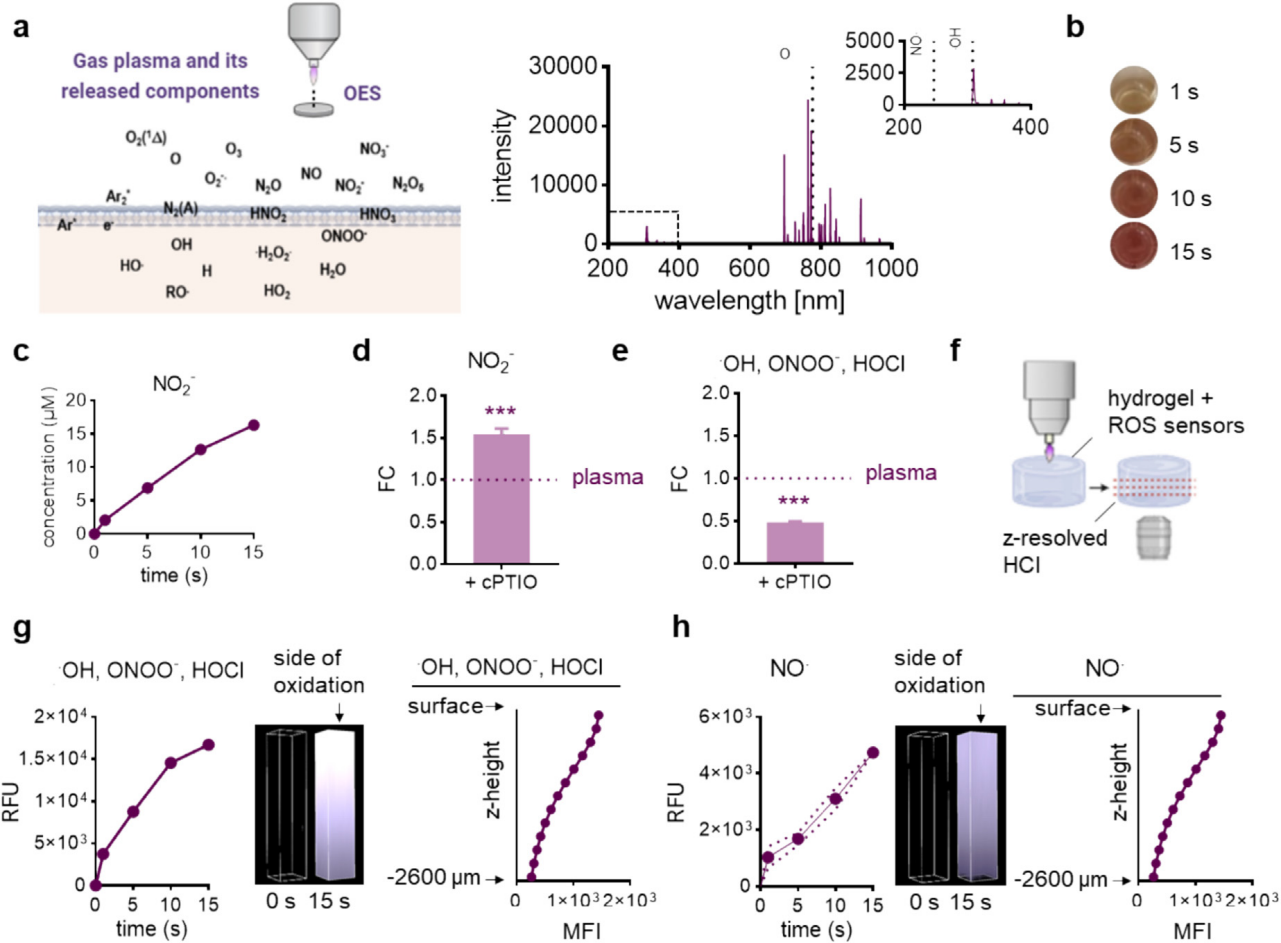
RNS profiling. (a) Optical emission spectroscopy (OES) of the kINPen Med plasma jet. (b) Representative images of the Griess reagent color change used for (c) quantification of nitrite (NO_2_^–^) in plasma-treated PBS. (**d-e**) Fold change (FC) of NO_2_^–^ (**d**), hydroxyl radical (^.^OH), peroxynitrite (ONOO^–^), and hypochlorous acid (HOCl, e); generated in PBS in the presence of the nitric oxide (NO^.^) scavenger carboxy-PTIO (cPTIO), normalized to plasma-treated samples without cPTIO. (f) Experimental setup for ROS profiling in 3D hydrogels. (**g**) Relative assessment of plasma-generated^.^OH, ONOO^–^ and HOCl in PBS (left) with representative 3D images of aminophenyl fluorescein fluorescence indicative of^.^OH, ONOO^–^ and HOCl in hydrogels after plasma treatment (middle) and their quantification (right graph). (**h**) Relative assessment of plasma-generated NO^.^ in PBS (left) with representative 3D images of diaminofluorescein fluorescence indicative of NO^.^ in hydrogels after plasma treatment (middle) and quantification thereof (right). Graphs show mean ± standard error of the mean (SEM). pl = plasma. O = atomic oxygen. RFU = relative fluorescence units. HCI = high content imaging. ROS = reactive oxygen species. MFI = mean fluorescence intensity. Data are expressed as mean ± S.D.; ***p < 0.001.

The formation of reactive species in the plasma gas phase was evaluated by optical emission spectroscopy (OES). Spectral lines at 247 nm, 308 nm, and 777 nm indicate the presence of nitric oxide (NO^.^), hydroxyl radicals (^.^OH), and atomic oxygen (O), among others, respectively (Fig. 2**a**). Due to their high reactivity, the evaluation of plasma-derived ROS deposition in biological systems is challenging, especially in complex systems such as tissues. In liquids, NO^.^, although it was not directly visible in OES, reacts rapidly with oxygen to form nitrite (NO_2_^–^), which was therefore first quantified as a surrogate marker (Fig. 2**b**), showing a treatment time-dependent generation (Fig. 2**c**). The addition of cPTIO was used as a scavenger for NO^.^, potentiating NO_2_^–^ generation in PBS (Fig. 2**d**). Likewise, the addition of cPTIO attenuated the generation of peroxynitrite (ONOO^–^), an oxidation product of NO^.^, as indicated by decreased fluorescence of the redox probe aminophenyl fluorescein (APF; Fig. 2**e**). Using embedded ROS sensors and z-resolved *high-content imaging*, the deposition of NO^.^ and secondary nitrogen species was then evaluated in hydrogel-based tissue models (Fig. 2**f**) after confirming their deposition in PBS. In PBS, a treatment time-dependent increase in APF fluorescence was observed, indicating increased ONOO^–^ generation (left graph, Fig. 2**g**). Z-resolved *high-content imaging* (image, Fig. 2**g**) further revealed an axial gradient of ONOO^–^ deposition compared to untreated hydrogels (right graph, Fig. 2**g**). Relative assessment of NO^.^ was performed using diaminofluorescein (DAF), which confirmed a time-dependent deposition in PBS (left graph, Fig. 2**h**) along an axial gradient in 3D hydrogels (image, Fig. 2**h**), as confirmed by algorithm-driven image analysis (right graph, Fig. 2**h**).

### Gas plasma-induced cytokine secretion alters inflammatory pattern to initiate healing

**Fig. 3:**
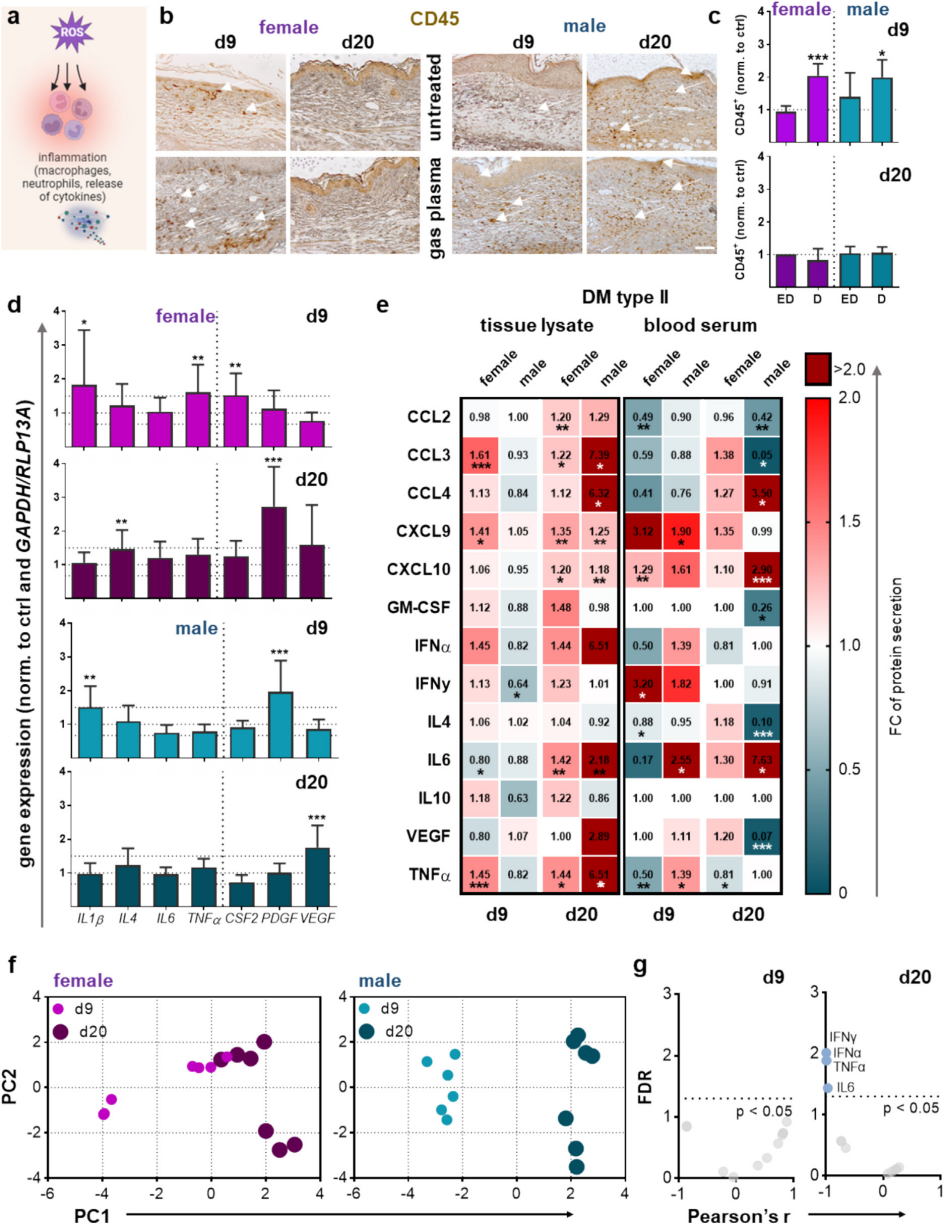

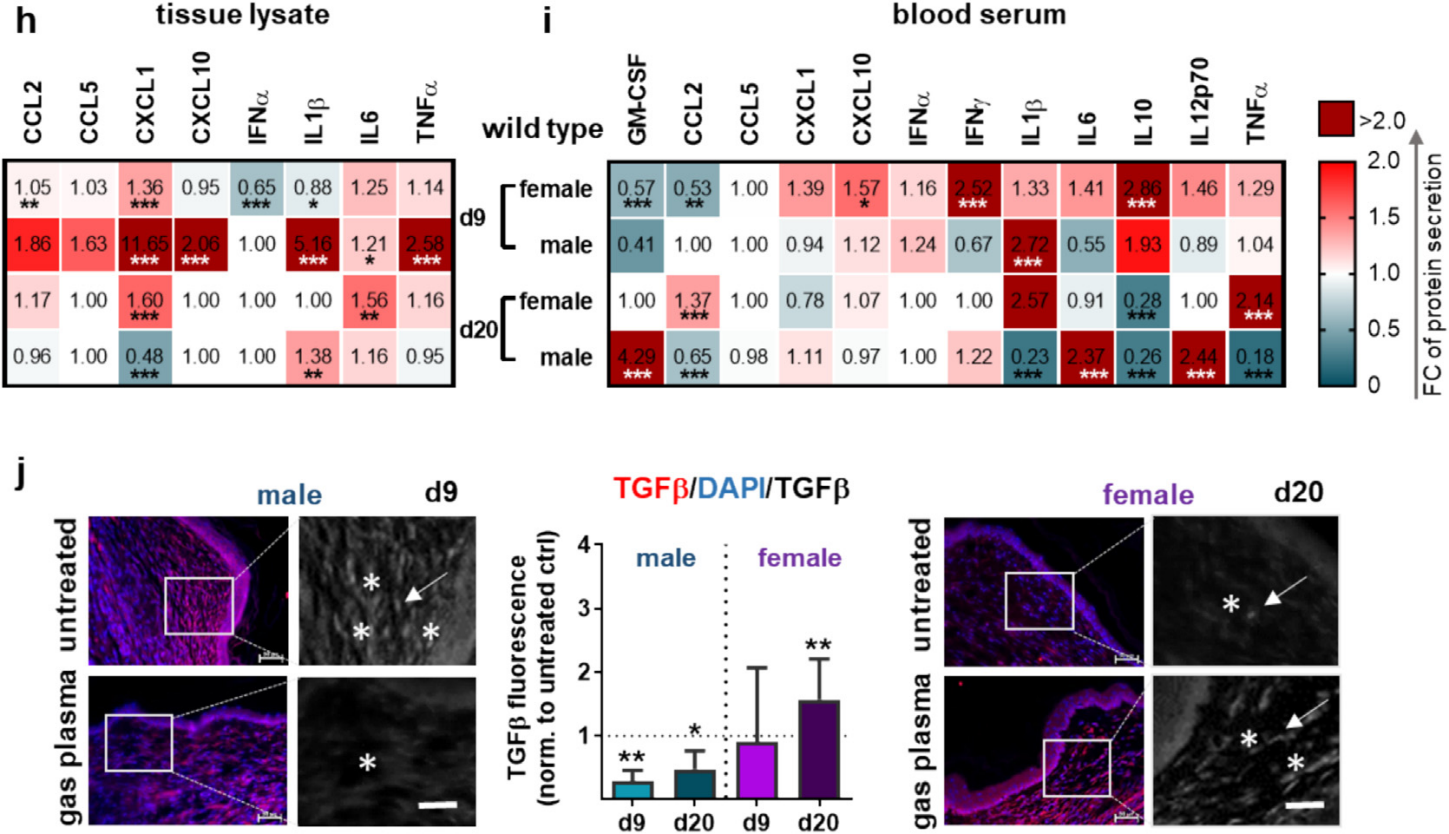
The pattern of inflammation is altered by gas plasma-induced cytokine secretion in diabetic wounds. (a) Effects of plasma-generated ROS on inflammation, cytokine, and chemokine release. (b) Representative immunohistochemical images of CD45 staining (some brown DAB signals indicated by arrows) in females (left) and males (right) at d9 and d20 after wounding are shown in plasma-treated compared to untreated diabetic wounds. (c) The number of CD45-positive cells in the wounds was counted at both endpoints after normalization to the total cell count and compared to untreated wounds. (d) qPCR-based expression analysis of interleukins (*IL1*β*/4/6*), tumor necrosis factor α (*TNF*α), and growth factors (*CSF2, PDGF, VEGF*) at both endpoints. (e) Heat maps show medians and indicate chemokine (CCL2/3/4, CXCL9/10), cytokine (IFNα/γ, IL4/6/10, TNFα), and growth factor (GM-CSF, VEGF) levels in tissue lysates (left) and in blood serum normalized to corresponding untreated control diabetic wounds. (f) Principal component analysis (PCA) of cytokine secretion levels in blood serum of diabetic mice. (g) Correlation of cytokine levels with wound closure measurements at d9 and d20. (h-i) Heat maps show medians and indicate cytokine (IFNα/γ, IL1β/6/10/12p70, TNFα), chemokine (IP10, KC, MCP1, RANTES), and growth factor (GM-CSF) levels in tissue lysates (h) or blood serum (i) normalized to corresponding untreated control (ctrl) wounds in the wild-type mouse model (C57BL/6J). (j) Immunofluorescence analysis of TGFβ staining and quantification (graph) at d9 in males (left) and at d20 in females (right). Data are expressed as mean ± S.D.; *p < 0.05, **p < 0.01, and ***p < 0.001 compared with untreated controls; scale bar is 50  µm; females and males were used at d9 and d20 (n ≥ 4). ED = epidermis; D = dermis.

Specific immune system responses are essential for proper progression through the various phases of wound healing (e.g., inflammation, proliferation, and remodeling). At the same time, modulation of inflammatory activity has potential therapeutic implications (Fig. 3**a**). Protein tyrosine phosphatase receptor type C (CD45) was used as a marker to show the influx of leukocytes (e.g., neutrophils, macrophages, T lymphocytes, fibrocytes) into the wound area. We found that gas plasma-treated wounds contained a higher number of CD45-positive cells (arrows) in females at d9 compared to other groups (left images, Fig. 3**b**). Conversely, CD45-expressing cells (arrows) were predominantly identified in males at d20 as shown in representative immunohistochemical staining (right images, Fig. 3**b**). However, quantification of CD45-positive cells (brown dots) showed a significant increase in CD45-expressing cells in dermal layers at d9 in both sexes compared to untreated tissue (top graph, Fig. 3**c**). The differences between plasma-treated and untreated wounds were only marginal at day 20 in both sexes (bottom graph, Fig. 3**c**). This finding suggests both a potentiation of the influx of specific CD45-expressing cells into wound areas and sex differences in the amount of CD45-positive cells at different stages of the wound healing.

The orchestrated expression of cytokines and chemokines is a complex and dynamic process that occurs both locally at the wound site and systemically throughout the body. Therefore, we next examined the expression of these factors in tissue lysates and blood. First, we performed qPCR analysis of several inflammatory cytokines, which showed a significant increase in the pro-inflammatory interleukin (*IL-1*β) in both sexes at d9. At the same time, tumor necrosis factor-alpha *(TNF*α*)* and (granulocyte–macrophage) colony-stimulating factor (*CSF2* transcript of GM-CSF) were upregulated in only diabetic females at both endpoints. *IL4* plays an essential role in regulating immune responses depending on the stage of wound healing and was moderately to significantly upregulated in the tissue remodeling and re-epithelialization phases, as shown here. *IL6* was slightly different in untreated and plasma-treated wound tissue. Both growth factors, the platelet-derived growth factor (*PDGF*), were higher in males at d9 and in females at d20; the vascular growth factor (*VEGF*) was mainly increased in both sexes at d20 (Fig. 3**d**).

It was evident that the local levels of cytokines and chemokines are mainly elevated earlier (d9) in plasma-treated females than in males (left heat map, Fig. 3**e**). It is also noteworthy that all expression levels, except for IFNγ (significantly lower) and, in some cases, IL10 (moderately lower), are unchanged in males at d9, and that substantial increases in some factors were observed only at the second endpoint. At d20, we found dramatically higher levels of several pro-inflammatory and other factors (e.g., CCL3/4, IFNα, TNFα, and VEGF) in males in contrast to females, where the expression was moderately increased. We confirmed the significant upregulation of IL6 in tissue lysates from both sexes only at d20, underscoring the balanced inflammatory response to prevent chronic inflammation and facilitate the transition to the proliferative phase. To understand whether gas plasma therapy has a systemic effect on pro- and anti-inflammatory factors and the immune response, we further mapped cytokine and growth factor secretion in blood serum (right heat map, Fig. 3**e**). It is noticeable that the differences at d9 (e.g.*,* increase of CXCL9, CXCL10, IFNγ and slight differences in levels of CCL2/3/4, IL4/10, GM-CSF, VEGF), are less pronounced between the two sexes. Levels of IFNα, IL6, and TNFα were increased only in males, suggesting that the initial inflammatory response was either already completed in females or that these levels were consistently low in females. At d20, we observed some differences in the release of CCL3, IL4, GM-CSF, and VEGF, all of which were significantly decreased in males, which in turn may explain the altered wound healing process in males.

To classify plasma-induced effects, we also mapped the inflammatory pattern in the C57BL/6J wild-type (Fig. 3**f**). In the absence of diabetes, we observed a switch of cytokine expression to upregulation (except for IFNα) in males on d9. In contrast, the levels in females were mainly unchanged (except for IFNα, IL1β, and CXCL1). At d20, the IL6 expression was increased in females, similar to the results above, as well as the chemokine CXCL1, whereas CXCL1 was significantly downregulated and IL1β considerably upregulated in males. In blood serum, all investigated cytokines and chemokines (except for CCL5) were differentially regulated after plasma treatment in females at d9. At the same time, only IL1β and IL10 were higher in the males of the wild-type strain. At the second endpoint (d20), we detected a differential regulation in both sexes, suggesting a shift in the expression and secretion patterns. GM-CSF levels were increased primarily in males, which correlates with its function as a critical cytokine being investigated as a potential therapeutic agent for chronic wounds (e.g., in diabetic and pressure ulcers) [35]. A critical mediator of the inflammatory response, IL1β, as well as CCL2 and TNFα levels were more pronounced in female wild-type mice (Fig. 3**g**). Cluster analysis of cytokine secretion levels in the blood serum of diabetic mice highlighted altered cytokine and chemokine release profiles at earlier and later time points, although differences where less pronounced in female mice (Fig. 3**h**). Cytokine levels were next correlated with wound closure measurements to identify targets associated with improved wound healing following plasma treatment. While no predictive response patterns were observed at d9, a significant negative correlation was observed for several pro-inflammatory mediators, i.e., IFNα, IFNγ, IL6, and TNFα, at d20 (Fig. 3**i**).

Next, we analyzed the expression pattern of transforming growth factor (TGF) β, a cytokine with multifaceted roles in immune regulation and either stimulatory or inhibitory properties in inflammation. Using immunofluorescence microscopy, TGFβ-positive areas were found in fibroblast-rich dermal layers at the wound site and in adjacent tissues. In males, quantification of TGFβ confirmed the findings of decreased release of TGFβ at d9 (left images). Its expression decreased to a similar extent at wound sites at d20 (graph, Fig. 3**j**). In female mice, during the early stages of wound healing on day 9, no clear difference was observed between the control and gas plasma-treated wounds until d20 when a slight increase was observed (right images, Fig. 3**j**).

### Gas plasma acts as Nrf2 activator in diabetic wounds

**Fig. 4:**
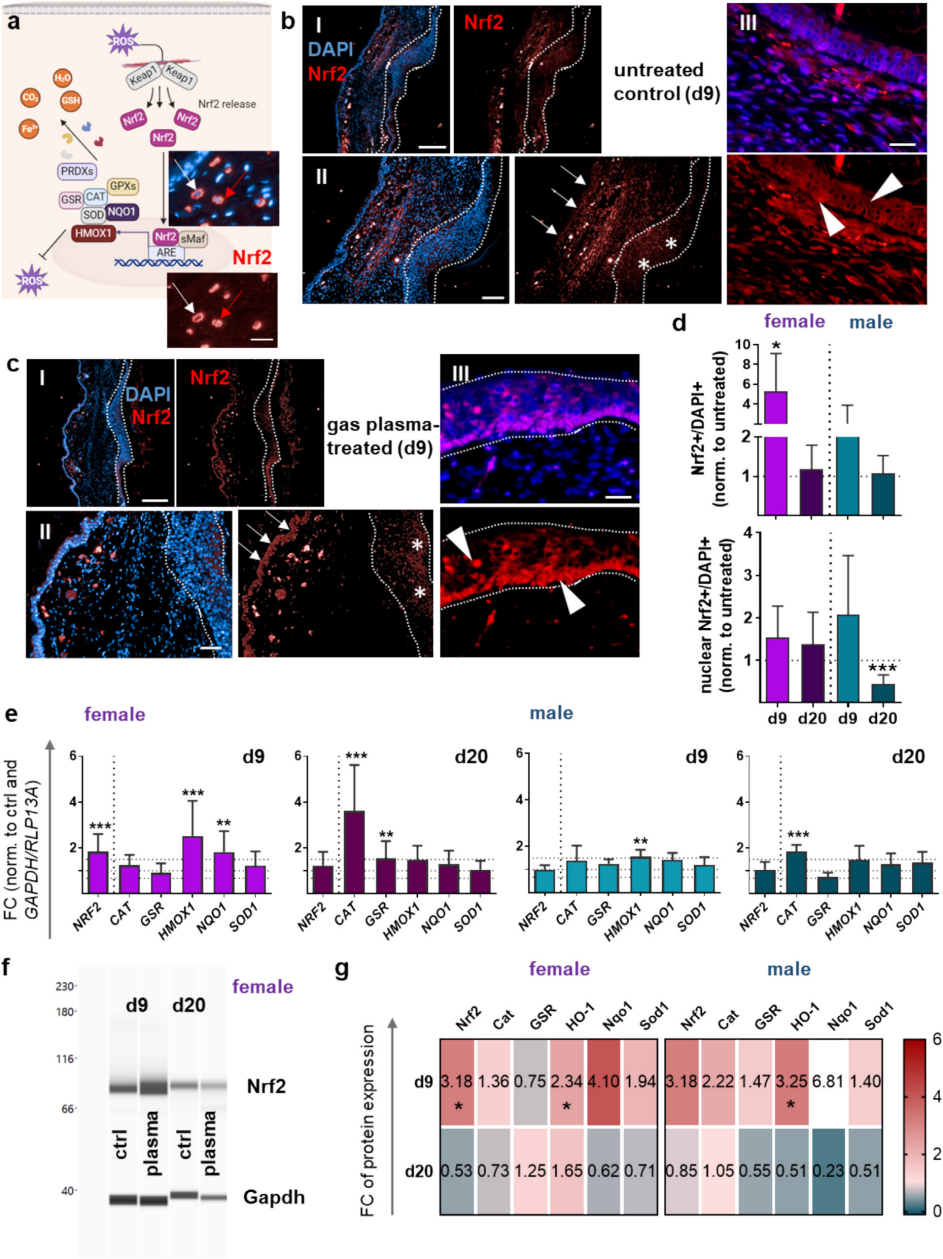
Gas plasma wound exposure promoted the expression of oxidative stress-induced targets. (**a**) Gas plasma-generated ROS affects Nrf2 signaling with nuclear translocation of the transcription factor Nrf2 (red arrows in fluorescence image) and ROS scavenging by activating Nrf2 signaling and the antioxidant defense system. (b-c) Distribution of Nrf2 in wound tissue after immunofluorescence staining on day 9 in untreated control (**b**) and after plasma treatment on day 9 (**c**) with 10-fold magnification (I), 20-fold magnification (II), and 40-fold magnification (III). Stars show Nrf2 staining at the wound site, arrows on the opposite side, and arrowheads show epidermal Nrf2 staining. (**d**) Quantification of Nrf2-positive cells in wound tissue (top graph, nuclear and cytoplasmic staining) and in nuclear staining of Nrf2 (bottom graph). (e) Quantification of mRNA expression of transcription factor *NRF2* and downstream targets (*CAT, GSR, HMOX1, NQO1, SOD1*). (**f**) Representative image of WES analysis of Nrf2 protein expression at d9 and d20 in females in plasma-treated wounds compared to untreated wounds (ctrl). Gapdh expression was used for housekeeping. (**g**) Nrf2, Cat, GSR, HO-1, Nqo1, and Sod1 protein expression levels were quantified by WES analysis at d9 and d20 in females (top panel) and males (bottom panel). Data are presented as mean ± S.D.; *p < 0.05, **p < 0.01, ***p < 0.001, as compared with controls (ctrl). Scale bars are 200 µm (top images), 100 µm (bottom images), and 20 µm (right images) in b, c.

Dysregulation of nuclear factor erythroid 2-related factor 2 (Nrf2) signaling is strongly associated with a diabetic phenotype [[36], [37]]. In light of a gas plasma-induced activation of Nrf2 signaling [32], we took a closer look at those in our diabetic mouse model to evaluate whether activation of stress-responsive pathways with gas plasma could improve diabetic wound healing. Under conditions of oxidative stress or redox modification (e.g., Keap1), free Nrf2 translocates to the nucleus, indicating a plasma-induced activation of the genes encoding cell-protective, antioxidant, and phase II detoxification enzymes (red arrow in fluorescence images) in contrast to normal homeostatic conditions (white arrow, Fig. 4**a**). Fluorescence microscopy of Nrf2 (red) is shown in representative figures at d9 in untreated (Fig. 4**b**) and plasma-treated wounds (Fig. 4**c**). Strong Nrf2 immunoreactivity was observed in the epidermis of plasma-treated wounds (stars (II), arrowhead in higher magnification (III), Fig. 4**c**), whereas Nrf2 was predominantly expressed in the cytoplasm of untreated wounds (stars (II), arrowhead in higher magnification (III), Fig. 4**b**). In addition, we identified an intense Nrf2 staining (arrows) on the opposite side of the plasma treatment (Fig. 4**cII**) compared to control (Fig. 4**bII**), suggesting paracrine plasma effects. Nrf2/DAPI-positive cells were quantified at both endpoints and showed an increase mainly at d9 in plasma-treated tissues compared to untreated controls (top graph, Fig. 4**d**). Nuclear Nrf2 staining in gas plasma-treated wounds, particularly in cells of the epidermal skin layers at d9, tended to be greater, except in males at d20 (bottom graph, Fig. 4**d**). Evidence of Nrf2 activation is reflected by an upregulation of the master regulator of antioxidant defense at d9 compared to untreated diabetic wounds as shown by qPCR (Fig. 4**e**) and WES analysis (Fig. 4**f-g**). This Nrf2 induction enhanced the coordinated expression of several Nrf2-regulated antioxidant response (ARE) genes that counteract oxidative stress. As expected, there was a concomitant substantial increase in the expression levels of catalase (*CAT*), heme oxygenase 1 (*HMOX1*, gene transcript of HO-1 protein), NAD(P)H quinone oxidoreductase 1 (*NQO1*), and superoxide dismutase 1 (*SOD1*) (with the exception of glutathione reductase (*GSR*) expression in females), together with an early (d9) and sustained activation at d20 in both sexes (Fig. 4**e**). A representative protein expression analysis using the WES system showed the increased Nrf2 expression after plasma treatment at d9, which was transient as indicated by lower expression at the d20 endpoint in females (Fig. 4**f**). In general, the overall protein expression levels decreased at d20, suggesting a transient induction of the antioxidant response (Fig. 4**g**). Finally, we determined the transcript levels of glutathione S-transferase A1/3 (*GSTA1/3*), Kelch-like ECH-associated protein 1 (*KEAP1*), peroxiredoxin 2 (*PRDX2*), and *SOD2,* which were significantly increased in females at d9, except for glutathione peroxidase 2 (*GPx2*). Transcript expression levels in males were similar to that of untreated controls at d9, confirming apparent sex-specific effects in gas plasma-exposed wound tissue. We found lower expression levels of these targets in diabetic wounds of both sexes at d20 (Fig. S1a).

**Fig. 5:**
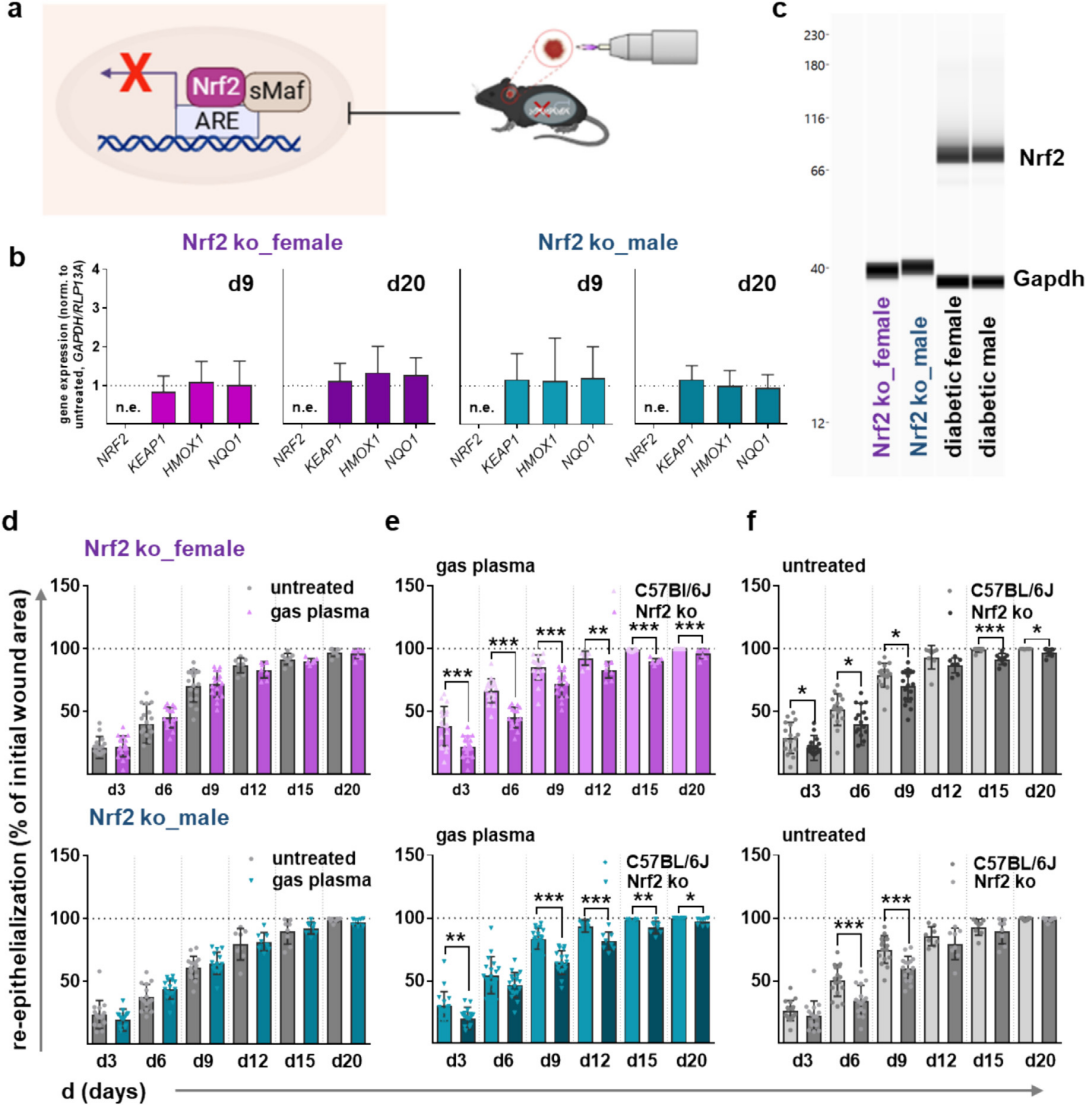
Gas plasma wound exposure affected the expression of oxidative stress-induced targets in an Nrf2 knockout mouse model. (**a**) Schematic of plasma treatment in a Nrf2 knockout mouse model. (**b**) Quantification of mRNA expression of the transcription factor *NRF2* and downstream targets (*KEAP1, HMOX1, NQO1*). (**c**) WES analysis of Nrf2 and Gapdh (housekeeping) protein expression in a Nrf2 knockout mouse model compared to diabetic mice in both sexes (pink, female; cyan, male). (**d**) The rate of wound closure (re-epithelialization) is plotted as the percentage reduction of the original wound area over time for gas plasma-treated Nrf2 knockout (ko) mice when compared to the untreated controls in females (pink, top panel) and males (cyan, bottom panel); (e) for gas plasma-treated Nrf2 ko mice when compared to C57BL76J mice, and (f) for untreated Nrf2 ko mice when compared to C57BL76J mice (n ≥ 4). For each group, individual scatters are displayed. Data are presented as mean ± S.D.; *p < 0.05, **p < 0.01, ***p < 0.001, as compared to controls (ctrl). Scale bar is 50 µm. n.e. = not expressed.

In addition, we asked whether gas plasma-induced signaling improves diabetic wound healing. Using an Nrf2 knockout (ko) mouse model with a deficiency of the master regulator of redox homeostasis during wound healing (Fig. 5**a**), Nrf2 ko correlated with an absence of *NRF2* expression as well as unchanged expression of downstream targets as determined by qPCR (Fig. 5**b**) and WES analysis for Nrf2 (Fig. 5**c, S1b**). More interestingly, we found a similar healing rate in plasma-treated and untreated Nrf2 ko wounds (Fig. 5**d**), supporting the re-epithelialization results along with a significantly lower epithelialization on each day and in both sexes compared to the wild-type strain (Fig. 5**e**). Similarly, wound closure rates were higher in untreated controls in non-diabetic compared to Nrf2 knockout mice (Fig. 5**f**). These results highlight that gas plasma-mediated responses through the Nrf2 pathway are strongly involved in cellular stress responses, antioxidant defense, and, ultimately, tissue repair and wound closure.

### Gas plasma-mediated changes in human wounds from diabetic patients

**Fig. 6:**
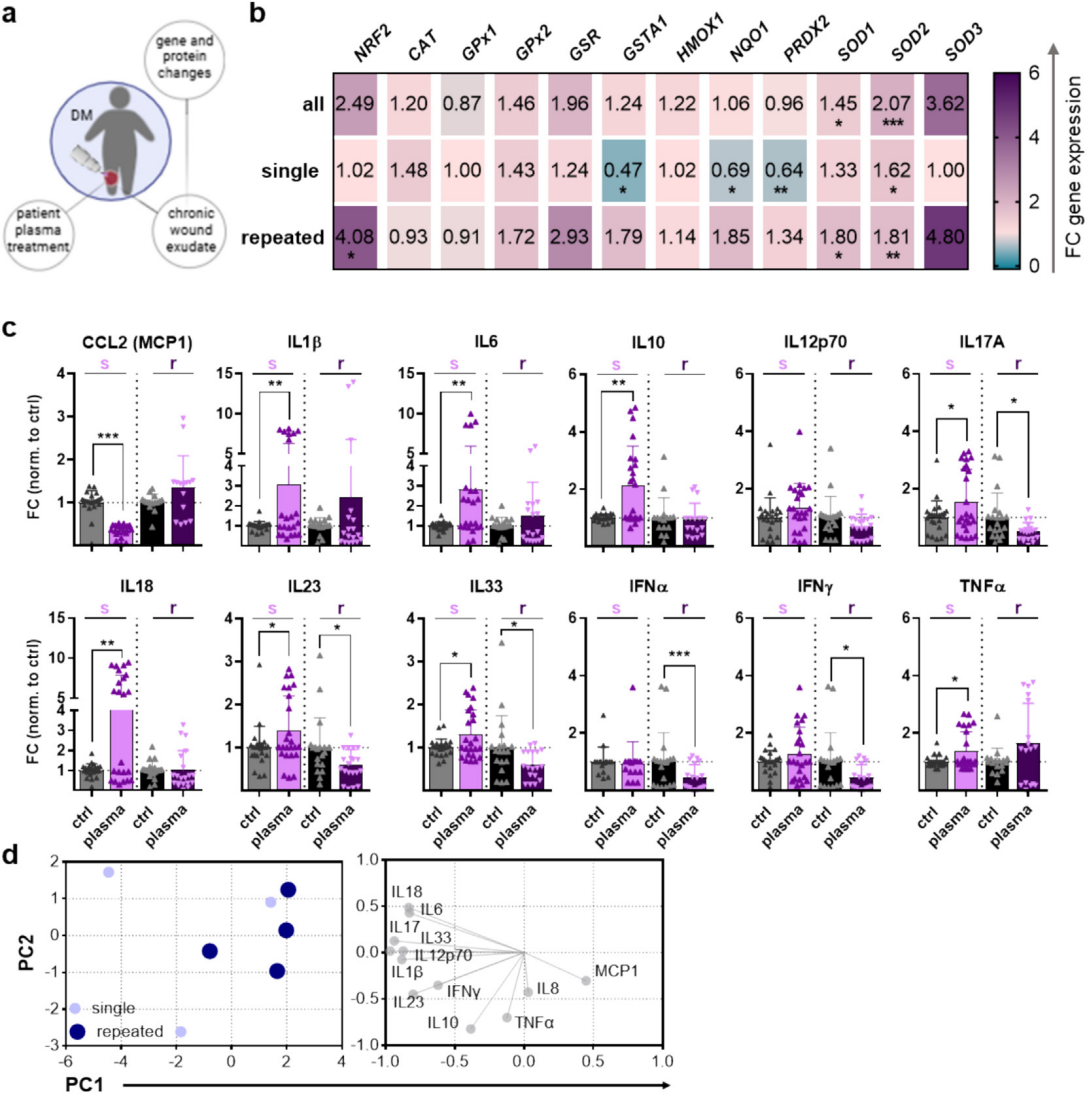
*In vivo* gas plasma treatment activated oxidative stress-induced targets such as Nrf2 signaling in diabetic patients. (**a**) Single (s) and repeated (r) gas plasma treatment of chronic wounds in diabetic patients. (b) Heat map of the quantification of the transcription factor *NRF2* itself and downstream targets (*CAT, GPx1/2, GSR, GSTA1, HMOX1, NQO1, PRDX2,* and *SOD1/2/3*) after single and repeated plasma treatment; the top row represents the average of both treatment regimens. (c) Bead-based quantification of cytokines such as CCL2 (MCP-1), IL-1β/6/10/12p70/17A/18/23/33, IFNα/γ, and TNFα in wound exudates from diabetic patients compared to untreated wound exudates (gray columns). (**d**) Principal component analysis (PCA) of cytokine secretion levels in wounds from diabetic patients after single (light blue circles) and repeated plasma treatment (dark blue circles). Data are presented as mean ± S.D.; *p < 0.05, **p < 0.01, ***p < 0.001, as compared to controls (ctrl).

Next, wounds from diabetic patients were exposed to gas plasma once or repeatedly and compared to untreated wound exudates from the same patients. The conductive mode of gas plasma treatment was used to enhance the delivery of reactive signaling molecules such as ROS directly to the tissue (Fig. 6**a**). Activation of the Nrf2 pathway may protect against inflammation, generally by reducing oxidative stress in diabetic tissues and by potentially reducing the complications associated with diabetes. Among the top upregulated targets was the Nrf2-stress signaling axis after repeated plasma exposure. Consistent with the increased expression and activity of Nrf2 as a transcription factor itself observed in our study, downstream targets such as HMOX1 (protein name HO-1), superoxide dismutases (SOD1/2/3), and glutathione reductase (GSR), which belong to the redox-sensitive proteins, were found to be highly upregulated even in response to repeated plasma treatment, as shown by qPCR (Fig. 6**b**) and bead-based cytokine analysis of wound exudates from diabetic patients (Fig. 6**c**). All pro-inflammatory cytokines such as CCL2 (MCP1, monocyte chemoattractant protein 1), IL1β, IL6, IL12p70, IL17A, IL18, IL23, IL33, IFNα/γ, and TNFα, recruit monocytes and macrophages to the wound site, and are critical in the early phases of wound healing. While IL10 is not directly involved in promoting inflammation, it is essential in wound healing, particularly in the later stages, to ensure proper resolution of inflammation and tissue repair. Following repeated plasma treatment (r), we have obtained a lower release of several pro-inflammatory interleukins such as IL1β, IL6, IL12p70, IL17A, IL18, IL23, IL33, IFNα/γ, in contrast to a single treatment (s), contributing to an anti-inflammatory environment that supports chronic wound healing. However, we found no changes in TNFα between single and repeated treatments but a higher release than in untreated wounds. CCL2 tended to be increased in some diabetic patients after repetitive gas plasma treatment. In diabetic wounds, reduced IL10 levels (anti-inflammatory cytokine), known to contribute to prolonged inflammation and delayed healing, were higher after single plasma treatment (Fig. 6**c**). Strikingly, principal component analysis of cytokine secretion levels in wounds from diabetic patients revealed dissimilar response patterns between patients after single exposure. In contrast, inflammatory mediators were consistently reduced after repeated plasma treatment, as shown by the principal component analysis (Fig. 6**d**).

## Discussion

Key factors driving the global type 2 diabetes epidemic include an aging population and rising obesity rates due to the availability of abundant food, sedentary lifestyles, environmental influences, and genetic predisposition [1]. Diabetes-related wounds exhibit a pronounced chronic disease course, high levels of oxidative stress, and suboptimal inflammatory responses, resulting in non-healing wounds and high wound care costs. Given the complexity and the need for an orchestrated coordination of the multistep wound healing process, the underlying mechanisms of gas plasma-mediated effects have rarely been investigated (reviewed in [[38], [39]]). Remarkably, only a handful of research groups have taken the step into the clinical application with plasma devices approved as medical devices for clinical use (reviewed in ^14^). In addition, there are still too few clinical and preclinical studies on gas plasma to better understand non-healing diabetic wounds [23,40]. To address this gap, we compared wound closure and activation of several signaling pathways involved in the healing process in preclinical applications of three different mouse models, including type II diabetes (B6.Cg-Lepob/J) and Nrf2 knockout (ko, B6.129X1-Nfe2l2tm1Ywk/J) compared to the wild-type mouse strain (C57BL/6J).

Why is the topical application of plasma-generated reactive species (ROS) of the novel gas plasma technology attractive for diabetic wound healing? Oxidative stress plays an essential role in the development and progression of both type I and type II diabetes [41]. The balance between antioxidant and pro-oxidant processes is inevitable through proper regulation of ROS production to ensure an optimal wound environment for efficient tissue repair. In addition, the regulation of cellular redox balance by ROS is involved in several stages of cellular and molecular wound healing processes, such as cell migration and proliferation and tissue remodeling [13] to restore skin integrity [42]. ROS are also involved in several redox-chemical pathways, such as inflammation [43], and play critical roles in orchestrating healing-related responses as secondary messengers for immune cells, as angiogenic mediators, and as players in host defense with bacteriostatic effects in the wound environment [[44], [45]]. Therefore, gas plasma offers several potential advantages over other treatments. Unlike some treatments that require injections or direct application of biochemical agents (such as recombinant growth factors), gas plasma is applied non-invasively. This minimizes patient discomfort and reduces the risk of additional tissue trauma or adverse reactions. Gas plasma devices can often be operated on an outpatient settings, with relatively low operating costs compared to the complex production and storage requirements of growth factor preparations. We have used medical gas plasma as a multi-component system that generates a variety of ROS in the gas phase [46] that are then transported directly to the defective tissue areas [47]. We confirmed a plasma-induced generation of reactive species such as nitric oxide (NO^.^), hydroxyl radicals (^.^OH), and atomic oxygen (O), among others (e.g., nitrite, peroxynitrite), as also shown previously or with other plasma devices [[48], [49]]. Considering that diabetes is characterized by a NO-deficient state associated with reduced collagen deposition and a severely impaired inflammatory response [50], gas plasma treatment of type II diabetic patients is more than reasonable.

Impaired wound healing is one of the most serious consequences of diabetes. It is associated with dysregulation of epithelialization in cellular compartments of the epidermis and an imbalance in extracellular matrix deposition in the dermis [7]. Accelerated re-epithelialization in acute wound models has been reported for an argon plasma jet [32,51] and other medical gas plasma devices using argon and/or helium [14,52]. Several reports have used preclinical rodent models to demonstrate plasma-induced healing (reviewed in [39], but few have used diabetic mice [[53], [54], [55], [56]]. Although most animal models that resemble human diabetes have limitations, the monogenic model of obesity with defects in leptin signaling (Lepob/ob) mirrors the human condition and has served as an established diabetes model for testing new therapies [[57], [58], [59], [60], [61], [62]]. Accordingly, our findings of reduced wound recovery rates and increased re-epithelialization after gas plasma exposure of diabetic wounds were demonstrated using this mouse model. However, the rate of re-epithelialization was much higher in the wild-type mouse strain, confirming previous reports of increased oxidative stress in the development and progression of diabetes [63]. However, diabetic wounds in females healed better than plasma-treated wounds in males, suggesting sex-specific differences. This finding is due to a combination of hormonal advantages and more efficient immune and tissue repair mechanisms. Estrogen has antioxidant properties that reduce the damage caused by free radicals, preventing delays in the healing process and resulting in better resistance to oxidative stress [64]. Higher estrogen levels in females are thought to enhance collagen production and the immune response, as shown here, and angiogenesis [32], whereas testosterone tends to suppress them in males [65]. An altered ratio of neutrophils to lymphocytes, which are markers of inflammatory processes (i.e., T-helper and T-killer cells), may cause differences in wound healing [66]. Strong evidence shows that the female immune system recognizes and fights pathogens more quickly [67]. These factors work together to promote faster recovery, less inflammation, and improved tissue regeneration in female mice compared to male mice, as shown here. Accelerated wound healing in female mice was accompanied by an early decrease in inflammatory mediators as early as d9, while the response patterns in male mice were more pronounced compared to later endpoints. However, hormonal differences could affect the oxidative stress response, cellular signaling pathways, and, ultimately, the repair processes that gas plasma treatment is designed to stimulate. The ability of estrogen to enhance antioxidant defenses may interact synergistically with the redox modulation induced by gas plasma, suggesting that treatment parameters (e.g., duration, intensity, frequency) may need to be adjusted between the sexes to achieve optimal results and to enhance the personalization of the treatment in clinical approaches.

Knowledge of gas plasma-induced signaling pathways *in vitro* in various epithelial and immune skin cells has contributed to understanding the relevance of gas plasma treatment in wound healing-related processes. Oxidative stress-sensitive signaling pathways, such as the nuclear factor Nrf2 pathway, are altered to regulate downstream target expression and cell behavior during wound healing [68]. However, dysregulation of Nrf2 signaling is strongly associated with a diabetic phenotype [[36], [37],^69^]. Essentially, Nrf2 levels are decreased in total cell extracts from diabetic wounds, and this abnormality appears to result from a diabetes-related decrease in Nrf2 protein stability [37]. Following gas plasma treatment, we demonstrated the activation of Nrf2 with its nuclear translocation *in vivo* at diabetic wound sites, which was associated with an increase in antioxidant capacity. To counteract the detrimental effects of ROS in diabetes, the antioxidant defense, which plays a critical role in maintaining redox balance to support optimal wound healing, was induced in our plasma-treated diabetic wound tissue. Thus, we demonstrated significantly increased expression of ARE genes, as previously shown [32,[70], [71]]. In wound exudates obtained from chronic wounds of diabetic patients, we showed that gas plasma treatment induced the expression of targets of the Nrf2 signaling pathway. Changes in superoxide dismutase (Sod), catalase (Cat), and glutathione peroxidase (GPx) levels were obtained, consistent with findings in plasma-treated diabetic rats [72]. In a wound healing assay that mimics re-epithelialization, gas plasma-induced activation of the Nrf2 pathway has been shown to promote skin cell migration, as demonstrated *in vitro* in HaCaT keratinocytes [73] and in co-culture models with fibroblasts [74]. Pharmacological activation of Nrf2 resulted in a more rapid reduction in wound diameter via increased expression of heme oxygenase 1 (HO-1) and NAD(P)H dehydrogenase [quinone] (Nqo) 1 [75]. Pharmacological activation of Nrf2 with the chemo-preventive dietary compounds sulforaphane (SF) and cinnamaldehyde (CA) also increased cell migration [26], demonstrating the importance of Nrf2 signaling. To investigate the mechanisms underlying wound healing, we applied gas plasma to ear wounds in an Nrf2 knockout mouse model and measured the rate of wound closure over time. Interestingly, we demonstrated decreased wound closure in Nrf2 knockout mice compared to the wild-type control, along with downregulated expression levels of genes related to oxidative stress. *In vivo* studies have shown that the rate of wound closure in STZ-induced diabetic Nrf2 knockout mice is slower than in the same wild-type mice because knockout of Nrf2 results in inadequate antioxidant signaling [75]. In addition to regulating antioxidant defenses, Nrf2 plays a multifaceted role in modulating inflammatory responses. Activation of Nrf2 leads to the expression of antioxidant enzymes that reduce intracellular ROS. ROS are key activators of inflammatory pathways, so their reduction indirectly attenuates the activation of pro-inflammatory transcription factors such as NF-κB [13]. Nrf2 has been shown to repress the transcription of specific cytokines and chemokines by modulating the chromatin environment around inflammatory gene promoters [76]. This crosstalk is essential for maintaining the balance between oxidative stress and inflammation. In immune cells, Nrf2 influences differentiation and cytokine production. For example, its activation in macrophages can lead to a more anti-inflammatory, or M2, phenotype, which is associated with tissue repair and resolution of inflammation [77].

Among the ROS generated by gas plasma, H_2_O_2_ stands out as the primary agent or the key mediator of Nrf2 activation [78]. It oxidizes cysteine residues on Keap1, which helps release Nrf2 to enter the nucleus and trigger the expression of antioxidant genes. While superoxide (O_2_•–) is also produced by gas plasma, it is rapidly converted to H_2_O_2_ by superoxide dismutase [79], meaning that its effects are channeled mainly through H_2_O_2_. In addition, nitric oxide (NO) and its derivative peroxynitrite (ONOO^−^) have been implicated in modulating redox signaling. They may further influence Nrf2 activation, contributing to redox signaling and wound repair [80]. In contrast, the hydroxyl radical (•OH) is exceptionally reactive and generally causes non-specific cellular damage rather than playing a controlled signaling role. However, more research is needed to fully dissect the individual roles of these reactive species in the context of gas plasma therapy.

What is the key link between the plasma-supported re-epithelialization and the switch from inflammation to the tissue formation and repair processes in diabetic wound healing? To answer this question, several lines of evidence in our study point to the modulation of key elements of inflammation, particularly in the early phases of wound healing. Diabetes is associated with chronic low-grade inflammation [81]. One strategy for treating non-healing wounds in diabetes is to find switches that directly target persistent inflammation and turn chronic wounds into acute ones [82]. Gas plasma appears to promote the ability to develop acute pro-inflammatory responses through the secretion of cytokines and chemokines. This apparent contradiction can be reconciled by considering that both the Nrf2 and NFκB pathways are part of a complex, context-dependent network of cellular responses to stress. First, gas plasma treatment can trigger an immediate stress response that rapidly releases cytokines and chemokines − an acute pro-inflammatory response −even as Nrf2 is activated. Over time, Nrf2 can help mitigate ongoing NFκB activity and chronic inflammation so that a regulatory, anti-inflammatory phase may follow the initial burst of inflammation. Second, the concentration of plasma-generated ROS may influence cellular outcomes. At certain plasma treatment times, plasma-induced oxidative stress may be sufficient to stimulate an acute inflammatory response via NFκB while simultaneously activating Nrf2 as a protective countermeasure. Thus, the overall response may initially appear pro-inflammatory before Nrf2-mediated suppression kicks in. Third, different cell types and tissue microenvironments may have different thresholds and regulatory mechanisms. In some contexts, gas plasma-induced oxidative stress may predominantly trigger NFκB-dependent cytokine secretion, whereas in others, Nrf2 activation may more effectively limit inflammation. Targeting cytokine signaling pathways (e.g., IL4/6, etc.) and inhibiting inflammatory factors may be beneficial in chronic wounds or excessive scarring conditions [83]. In the proliferative phase, IL6 promotes the proliferation and migration of keratinocytes and fibroblasts, influencing the production and remodeling of collagen and other ECM components [83]. CD45-positive cells (e.g., fibrocytes and leukocytes), which also regulate inflammation, immune responses, and cell signaling and contribute to tissue repair and remodeling [84], were transiently upregulated, which together ensure that the wound healing process is efficient and results in the restoration of tissue integrity. In healthy, type I, and type II diabetic rats, accelerated wound closure, neovascularization, and reduced neutrophil and T-cell distribution in diabetic wounds were found in gas plasma-treated tissues [72,85]. Macrophages expressing the leukocyte common antigen CD45 play a role in this phase by regulating the balance between collagen synthesis and degradation to ensure that proper tissue architecture is restored [86]. Overall, the data indicated a modest to significant effect of repeated gas plasma treatment on the cytokine signature in diabetic mice. In addition to minor differences between qPCR expression data and expression in tissue lysates, we found much larger differences between the two measurement endpoints and between local and systemic responses. Similar and different trends were also found between females and males, suggesting sex differences in regulating systemic and local cytokine release in response to plasma treatment. The transient increase in cytokines after a single treatment (e.g., IL17A or IL23) in patient samples appears to be part of the normal initiation of the healing process, while the decrease after multiple treatments suggests a transition to a regulated, anti-inflammatory environment that supports long-term tissue repair.

There are many challenges associated with oxidative stress-based therapies, such as gas plasma-induced treatment of chronic wounds. While the current study suggests an association between Nrf2 activation and improved wound healing, further experiments using Nrf2 inhibition in wild-type mice would be critical to confirm whether Nrf2 is indeed required for these healing effects. If pharmacological inhibition of Nrf2 in wild-type mice results in reduced healing, this would strongly support the idea that Nrf2 is critical for mediating the beneficial effects of plasma treatment. Nevertheless, knockout mice provide a robust and unambiguous model to dissect the mechanistic role of Nrf2 in gas plasma-induced wound healing, eliminating many of the potential confounding factors inherent in pharmacological approaches. Knockout mice completely lack the Nrf2 gene, ensuring that there is no residual Nrf2 activity. In contrast, pharmacological inhibitors may not completely suppress Nrf2 due to limitations in dosage, bioavailability, or inherent partial inhibition. Genetic knockout eliminates concerns about off-target effects that may be associated with small molecule inhibitors. These off-target effects can confound the interpretation of experimental results by affecting other pathways. In knockout mice, the absence of Nrf2 is consistent and stable throughout development and adulthood. This consistency can reduce variability and allow a more explicit interpretation of how Nrf2 absence affects wound healing and response to gas plasma treatment.

Determining optimal treatment parameters for gas plasma therapy remains an area of research, and there is no one-size-fits-all approach. Optimization of these parameters is critical for translating laboratory findings into effective clinical protocols. Our approach will assist in developing standardized treatment guidelines that may improve patient outcomes in diabetic wound care. Consistent with previous findings [[33], [34]], we suggest an optimal treatment duration of 5 to 10 s/cm^2^ and a working distance between the plasma source and the tissue of approximately 1 cm^2^ in preclinical studies. The number of treatments and the interval between them often depend on the specific clinical application. Future studies should focus on experimental designs that manipulate these variables independently and in combination, possibly using pilot clinical trials to establish treatment time-response relationships. However, we have shown that repeated gas plasma treatment enhances the antioxidant response, suggesting a shift from a chronic to an acute wound environment. In addition, gas plasma treatments are generally considered safe, especially in applications such as wound healing [31] and dermatology [14,87]. Plasma-generated ROS are highly transient and act locally, reducing the likelihood of systemic side effects. Their short lifetime means they do not accumulate in tissues and cause significant damage to healthy tissue. While current clinical experience suggests minimal side effects such as temporary local discomfort, redness, or mild irritation immediately after treatment, comprehensive long-term studies are still limited [88]. Therefore, long-term studies in chronic wounds are needed in clinical trials.

## Conclusion

The study highlighted the molecular consequences of gas plasma treatment in relevant preclinical wound models such as diabetes, where stress-responsive signaling pathways are affected. Here, we investigated the local effects of repeated gas plasma administration in a monogenic model of obesity and diabetes. First, our results elucidate gas plasma-promoted wound closure in diabetic wounds. Second, we used gene and protein expression analysis to observe that topical wound treatment with gas plasma-derived reactive species induced a redox-mediated stress response in diabetic skin tissue. Third, we demonstrated that the redox-mediated effects of gas plasma through Nrf2 signaling block prolonged inflammation in diabetic wound healing. These therapeutic effects of gas plasma were substantiated in an established Nrf2 knockout as a relevant model of Nrf2 deficiency. In this model, we observed that topical application of gas plasma has no supportive effects on wound closure, including epithelialization, but alters the cytokine and chemokine expression profile compared to the wild-type strain. Finally, we demonstrated in diabetic patients that the Nrf2 signaling pathway was activated after repeated gas plasma treatment, confirming preclinical observations. Thus, the protective role of Nrf2 in the context of diabetes mellitus by mitigating oxidative stress represents a promising target for developing new treatments to reduce diabetic complications. Our observations − if applicable to human wounds − provide novel insights into the gas plasma-induced mechanism of diabetic wound healing.

## Funding

This work was partly sponsored by the German Federal Ministry of Education and Research (BMBF, to SB, grant numbers 03Z22DN11 and 03Z22Di1). The funders had no role in study design, data collection, data analysis, manuscript preparation, and/or publication decisions.

## Declaration of competing interest

The authors declare that they have no known competing financial interests or personal relationships that could have appeared to influence the work reported in this paper.
