# Supplementary material for: Medical gas plasma modifies Nrf2 signaling in diabetic wound healing
Source: J Adv Res. 2025 Apr 16;80:179–95. doi: 10.1016/j.jare.2025.04.020 (PMC12869232; doi:10.1016/j.jare.2025.04.020)

Supplemental data

Table S1. Murine gene-specific primers used in qPCR.

| **Gene name** | **Gene ID** | **Primer sequences (3`- 5`)** |
| --- | --- | --- |
| nuclear factor erythroid 2-related factor 2 | *NRF2* | GAG TCG CTT GCC CTG GAT ATC  TCA TGG CTG CCT CCA GAG AA |
| heme oxygenase 1 | *HMOX1* | TGA AGC AGG CAT CTG AGG G  CGA AGG TGG AAG AGT GGG AG |
| Kelch-like ECH-associated protein 1 | *KEAP1* | CGGGGACGCAGTGATGTATG  TGTGTAGCTGAAGGTTCGGTTA |
| NAD(P)H dehydrogenase [quinone] 1 | *NQO1* | GGC ATC CAG TCC TCC ATC AA  GTT AGT CCC TCG GCC ATT GTT |
| catalase | *CAT* | CAG AGA GCG GAT TCC TGA GAG A  CTT TGC CTT GGA GTA TCT GGT GAT |
| superoxide dismutase [Cu-Zn] | *SOD1* | GAA ACA AGA TGA CTT GGG CAA AG  TTA CTG CGC AAT CCC AAT CA |
| superoxide dismutase, mitochondrial | *SOD2* | GTC CGA TGA TGT CAG CCA  AAC CCA TTT GCC GCT ACT |
| glutathione-disulfide reductase | *GSR* | TCG GAA TTC ATG CAC GAT CA  GGC TCA CAT AGG CAT CCC TTT |
| glutathione S-transferase A1 | *GSTA1* | CAG CCT GGC AGC CAG AGA  TCT GTG GCT CCA TCA ATG CA |
| glutathione S transferase alpha 3 | *GSTA3* | GGT TCC TGG TTT GTT CCT TG  CTA TGG GAA GGA CAT GAA GGA G |
| peroxiredoxin 2 | *PRDX2* | GGT GCC TTC AAG GAA ATC AA  GCC TAG CTT TCG GAA GTC CT |
| colony-stimulating growth factor | *CSF2* | GAC CCA ACT ATG ATG CGA GCC  CCC ATC CCA CAG GTC TTA GAA C |
| vascular endothelial growth factor | *VEGF* | AAC GAT GAA GCC CTG GAG TG  GAC AAA CAA ATG CTT TCT CCG |
| platelet-derived growth factor | *PDGF* | ATG AGA GTG AGA TCG AAGG CA  CGG CAAG GTA TGA TGG CAG AG |
| interleukin 1β | *IL1β* | GCA ACT GTT CCT GAA CTC AAC T  ATC TTT TGG GGT CCG TCA ACT |
| interleukin 4 | *IL4* | GGT CTCA ACC CCC AGC TAG T  GCC GAT GAT CTC TCT CAA GTG AT |
| interleukin 6 | *IL6* | ATC CAG TTG CCT TCT TGG GAC TGA  TAA GCC TCC GAC TTG TGA AGT GGT |
| tumor necrosis factor-alpha | *TNFA* | TCT CAT GCA CCA CCA TCA AGG ACT  ACC ACT CTC CCT TTG CAG AAC TCA |
| tumor necrosis factor beta 1 | *TGFB1* | TTT GGA GCC TGG ACA CAC AGT ACA  TGT GTT GGT TGT AGA GGG CAA GGA |
| glyceraldehyde 3-phosphate dehydrogenase | *GAPDH* | CAT GGC CTC CAA GGA GTA AG  TGT GAG GGA GAT GCT CAG TG |
| ribosomal protein 13A | *RLP13A* | AGC CTA CCA GAA AGT TTG CTT AC  GCT TCT TCT TCC GAT AGT GCA TC |

Table S2. Human gene-specific primers used in qPCR.

| **Gene name** | **Gene ID** | **Primer sequences (3`- 5`)** |
| --- | --- | --- |
| nuclear factor erythroid 2-related factor 2 | *NRF2* | AGG ACC CAG AAG CGC ACA TGA G  GGA TGT GCT GGG CTG GCT GAA T |
| catalase | *CAT* | TTT CCC AGG AAG ATC CTG AC  ACC TTG GTG AGA TCG AAT GG |
| glutathione peroxidase 1 | *GPx1* | CAG TCG GTG TAT GCC TTC TCG  GAG GGA CGC CAC ATT CTC G |
| glutathione peroxidase 2 | *GPx2* | CGG GGC TCA CTC TGC GCT TC  AGT CCC GGG TGG TTG TGC CT |
| glutathione-disulfide reductase | *GSR* | CGG CCG CAG CGT CAT TGT TG  CGC CAG CGT TCT CCA GCT CC |
| glutathione S-transferase A1 | *GSTA1* | CTG CCC GTA TGT CCA CCT G  AGC TCC TCG ACG TAG TAG AGA |
| heme oxygenase 1 | *HMOX1* | GGC CTG GCC TTC TTC ACC TT  GAG GGG CTC TGG TCC TTG GT |
| NAD(P)H dehydrogenase [quinone] 1 | *NQO1* | GGG CAA GTC CAT CCC AAC TG  GCA AGT CAG GGA AGC CTG GA |
| peroxiredoxin 2 | *PRDX2* | CCA GAC GCT TGT CTG AGG AT  ACG TTG GGC TTA ATC GTG TC |
| superoxide dismutase [Cu-Zn] | *SOD1* | ACT GGT GGT CCA TGA AAA AGC  AAC GAC TTC CAG CGT TTC CT |
| superoxide dismutase, mitochondrial | *SOD2* | GCT CCG GTT TTG GGG TAT CTG  GCG TTG ATGTGAGGTTCCAG |
| superoxide dismutase 3 | *SOD3* | ATG CTG GCG CTA CTG TGT TC  CTC CGC CGA GTC AGA GTT G |
| insulin growth factor 1 | *IGF1* | GCT CTT CAG TTC GTG TGT GG  GCC TCC TTA GAT CAC AGC TCC |
| insulin growth factor 2 | *IGF2* | GTG GCA TCG TTG AGG AGT G  CAC GTC CCT CTC GGA CTT G |
| β actin | *BACT* | GAG AGG GAA ATC GTG CGT GA  ACA TCT GCT GGA AGG TGG AC |
| smooth muscle actin α | *aSMA* | AAA AGA CAG CTA CGT GGG TGA  GCC ATG TTC TAT CGG GTA CTT C |
| collagen 1A1 | *COL1A1* | GTG CGA TGA CGT GAT CTG TGA  CGG TGG TTT CTT GGT CGG T |
| keratin 1 | *KRT1* | AGA GTG GAC CAA CTG AAG AGT  ATT CTC TGC ATT TGT CCG CTT |
| glyceraldehyde 3-phosphate dehydrogenase | *GAPDH* | AGG GCT GCT TTT AAC TCT GGT  CCC CAC TTG ATT TTG GAG GGA |
| ribosomal protein 13A | *RLP13A* | GCC CTA CGA CAA GAA AAA GCG  TAC TTC CAG CCA ACC TCG TGA |

Table S3. Patient samples and clinical characteristics. HbA1c = glycated hemoglobin value; s = single plasma treatment; r = repeated plasma treatment; x = unknown (anonymized).

| **ID** | **sex** | **age** | **HbA1c (%)** | **wound type** | **~size of wound (cm**^2^**)** | **plasma** | **analysis** |
| --- | --- | --- | --- | --- | --- | --- | --- |
| 1 | m | **76** | 5.5 | ulcus | 136 | s/ r (>5)  /repeated | qPCR |
| 2 | m | **86** | 6.3 | ulcus | 159 | s/r (>5) | qPCR |
| 3 | m | **82** | 7.9 | ulcus | 1 | s | qPCR |
| 4 | m | 60 | 7.5 | ulcus | 48 | 0 | qPCR |
| 5 | w | 70 | x | deep ulcus | 209 | r (>5) | cytokines |
| 6 | w | 70 | x | deep ulcus | 132 | r (>5) | cytokines |
| 7 | w | **89** | x | ulcera crurus mixta | 12 | r (>5) | qPCR, cytokines |
| 8 | m | **78** | x | deep ulcus | 9 | r (<5) | qPCR, cytokines |
| 9 | w | 70 | x | deep ulcus | 132 | r (>5) | cytokines |
| 10 | m | **59** | x | unknown | 3 | s | qPCR, cytokines |
| 11 | w | 66 | x | ulcus | 5 | r (<5) | cytokines |
| 12 | w | 70 | x | deep ulcus | 209 | r (>5) | cytokines |
| 13 | m | **84** | x | ulcus mixtum | 8 | s | qPCR, cytokines |

Figure S1. Gas plasma treatment **modulates the** expression of **oxidative stress-responsive targets** and analysis of **Nrf2 expression in the knockout mouse model**. (a) qPCR-based expression analysis of several oxidative stress-responsive targets (*GPx2, GSR, GSTA1/3, PRDX2, SOD2)* with total RNA isolated from the wound regions at both endpoints (d9/d20) in diabetic female (left) and male (right) mice. (**b**) Protein expression analysis of Nrf2 and Gapdh was performed with protein lysate samples of female and male wounds in an Nrf2 ko mouse model compared to a wild-type mouse model. (**c**) Protein expression analysis of Sod1, Cat, and Gapdh (representative for d9) was performed with protein lysate samples from female and male wounds in a diabetic mouse model. (**d**) Protein expression analysis of HO-1, Nqo1, and β actin (representative for d9, d20) was performed with protein lysate samples from female and male wounds in a diabetic mouse model. (**d**) Protein expression analysis of GSR (representative for d9, d20) was performed with protein lyses samples of female wounds in a diabetic mouse model. Data are presented as mean ±S.D.; *p<0.05, and **p<0.01 compared to controls; females and males were used at d9 and d20 (n > 4).


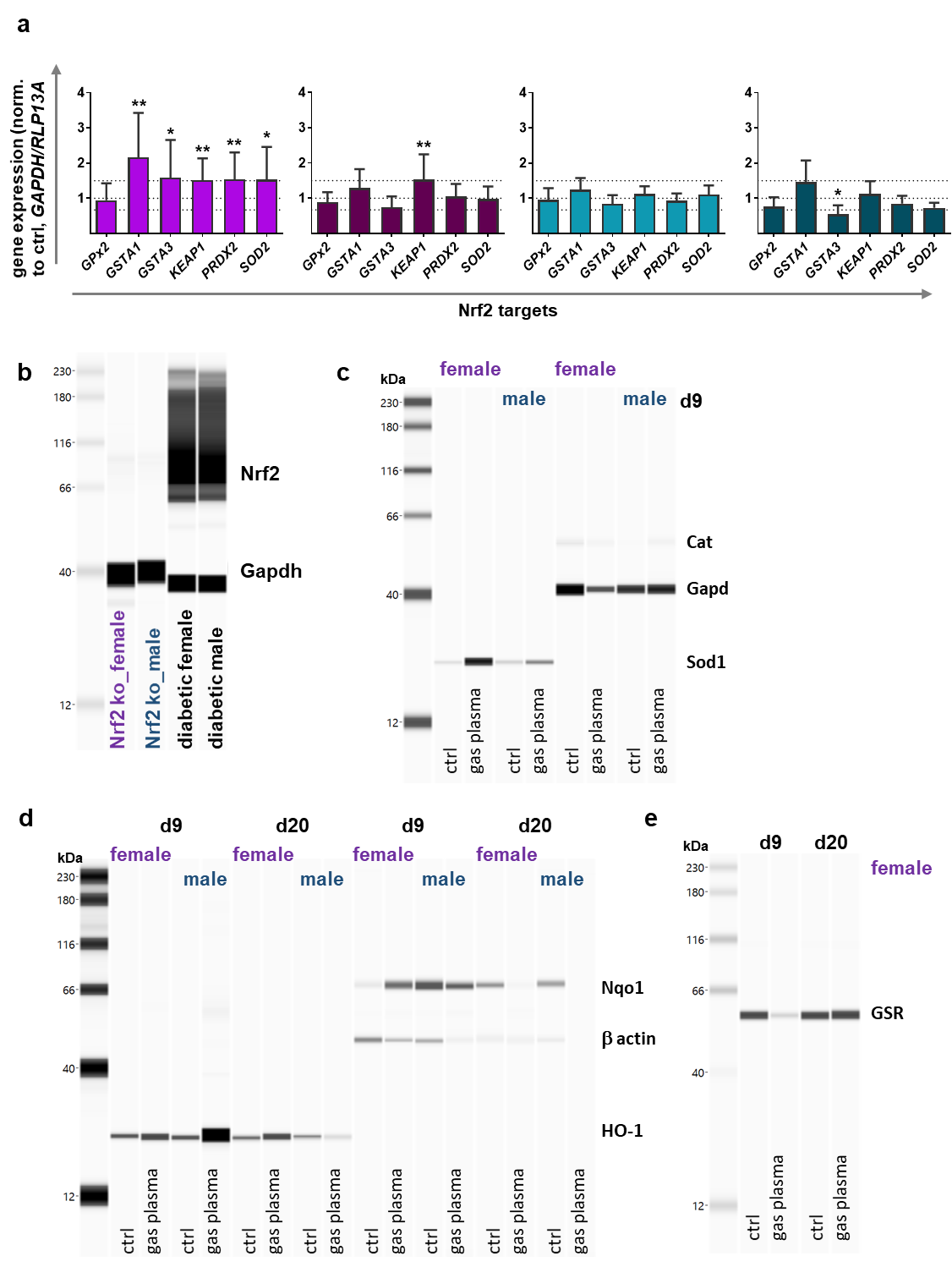

Supplement: Supplementary Data 1 [file mmc1.docx]
